# Proteomic Analysis of the Excretory and Secretory Proteins of *Haemonchus contortus* (HcESP) Binding to Goat PBMCs *In Vivo* Revealed Stage-Specific Binding Profiles

**DOI:** 10.1371/journal.pone.0159796

**Published:** 2016-07-28

**Authors:** Javaid Ali Gadahi, Shuai Wang, Gao Bo, Muhammad Ehsan, RuoFeng Yan, XiaoKai Song, LiXin Xu, XiangRui Li

**Affiliations:** College of Veterinary Medicine, Nanjing Agricultural University, Nanjing, 210095, PR China; Macquarie University, AUSTRALIA

## Abstract

*Haemonchus contortus* is a parasitic gastrointestinal nematode, and its excretory and secretory products (HcESPs) interact extensively with the host cells. In this study, we report the interaction of proteins from HcESPs at different developmental stages to goat peripheral blood mononuclear cells (PBMCs) *in vivo* using liquid chromatography-tandem mass spectrometry. A total of 407 HcESPs that interacted with goat PBMCs at different time points were identified from a *H*. *contortus* protein database using SEQUEST searches. The L_4_ and L_5_ stages of *H*. *contortus* represented a higher proportion of the identified proteins compared with the early and late adult stages. Both stage-specific interacting proteins and proteins that were common to multiple stages were identified. Forty-seven interacting proteins were shared among all stages. The gene ontology (GO) distributions of the identified goat PBMC-interacting proteins were nearly identical among all developmental stages, with high representation of binding and catalytic activity. Cellular, metabolic and single-organism processes were also annotated as major biological processes, but interestingly, more proteins were annotated as localization processes at the L_5_ stage than at the L_4_ and adult stages. Based on the clustering of homologous proteins, we improved the functional annotations of un-annotated proteins identified at different developmental stages. Some unnamed *H*. *contortus* ATP-binding cassette proteins, including ADP-ribosylation factor and P-glycoprotein-9, were identified by STRING protein clustering analysis.

## Introduction

*Haemonchus contortus* (*H*. *contortus*) is the most important abomasal nematode of small ruminates. *H*. *contortus* infection causes high economic losses worldwide [1, 2]. This worm penetrates the abomasal mucosa to feed on the blood of the host, resulting in anemia and low total plasma protein [3, 4]. *H*. *contortus* is one of the most extensively used parasitic nematodes in drug discovery, vaccine development and anthelmintic resistance research [5–8]. The development of first (L_1_), second (L_2_) and third (L_3_) stage *H*. *contortus* larvae occurs in the faeces. The infective larvae (L_3_) are ingested by the host with herbage. After exsheathment triggered by pepsin and HCl in the rumen, L_3_ migrates to the abomasum and develops into the L_4_ stage, which feeds on blood, followed by final development into adults approximately 3 weeks post ingestion [9]. Each developmental stage has different motility, sensory and hormonal regulation requirements, which may require rapid transcriptional changes [10].

Excretory and secretory products (ESPs) are produced and released by parasites during *in vitro* cultivation [11] and *in vivo* [12]. ESPs contain various proteins and glycoproteins whose functions include depression of host immunity and modulate the host immune system from the early stages of infection for their survival [13–15].

*H*. *contortus* excretory and secretory products (HcESPs) contain many proteins [16] that perform diverse functions such as tissue penetration and host protein degradation [14]. A 55 kDa secretory glycoprotein was identified as an immunogenic protein that causes immune modulation by inhibiting host neutrophils [17]. The purified 66 kDa adult *H*. *contortus* excretory/secretory (E/S) antigen inhibits monocyte function *in vitro*, as confirmed by decreased production of hydrogen peroxide and nitric oxide in the culture medium [11]. One HcESP protein induces eosinophil and neutrophil chemotactic activity [18]. We have also demonstrated that recombinant *H*. *contortus* galectin (rHco-gal-m) is recognized by the serum of goats naturally infected with *H*. *contortus* and can bind and modulate the activity of goat T cells and monocytes. rHco-gal-m inhibits the expression of MHC II molecules, decreases T cell activation and proliferation, induces the apoptosis of T cells and affects several signaling cascades [19]. *In vitro* studies have reported that parasitic ESPs have a direct effect on cultured cells or tissues, such as inhibiting acid secretion [20] and inducing the vacuolation and detachment of HeLa cells [21, 22]. These findings indicate that ESPs have multiple functions *in vivo*.

In a previous analysis of HcESP, approximately 193 immunogenic spots were detected by 2D gel analysis [16], and 52 proteins were identified by MS. We identified 129 male-specific, 132 female-specific and 23 shared immunogenic proteins from adult *H*. *contortus* by MALDI-TOF [23]. The presence of antibodies against many E/S proteins in infected animals strongly indicates the presence of ESPs in the circulation of infected animals [12, 23]. Other intestinal nematodes of livestock that are very closely related to *H*. *contortus*, including *Cooperia* spp. [24], *Ostertagia ostertagi* [25], and *Teladorsagia circumcincta* [26], also secrete a GAL/VAL-dominated suite of ESPs. The large number of ESP molecules also suggests functional complexity.

Binding to the host cell is often a prerequisite for ESP function [11, 17–19, 27]. Some ESP molecules react to the molecules on the surface of the host cell to form receptor-ligand complexes, similar to many other receptor-ligand systems, for example, galectin binds β-galactoside sugars in a metal-independent manner [28, 29].

Despite the large number of ESP molecules and their diverse functions, few ESP proteins have been identified and functionally characterized, particularly *in vivo*, and the ESP receptors on the host cell surface have not been fully characterized. Peripheral blood mononuclear cells (PBMCs) consist of several populations of immune cells, included lymphocytes (T cells, B cells, and NK cells) and monocytes that play important roles in the immune responses. Previously we reported that, HcESPs had immune suppressive potential on the goat PBMCs *in vitro* [30]. The present study is the first to analyze HcESPs from different developmental stages of *H*. *contortus* that interact with goat PBMCs *in vivo* using proteomics. This study will facilitate the elucidation of HcESP functions and the mechanisms of *H*. *contortus* immune evasion and pathogenesis.

## Materials and Methods

### Ethics Statement

Animal experiments were conducted following the guidelines of the Animal Ethics Committee, Nanjing Agricultural University, China. All experimental protocols were approved by the Science and Technology Agency of Jiangsu Province. The approval ID is SYXK (SU) 2010–0005.

### Production of *H*. *contortus* excretory and secretory product (HcESP) *in vitro*

To harvest ESP, the standard procedure for *H*. *contortus* described by Yatsuda et al. was used [16]. Briefly, *H*. *contortus* (Nanjing strain) adult worms were harvested from the abomasum of an experimentally infected donor goat, washed several times with PBS, and incubated for 4 h in RPMI 1640 medium (100/ml) containing antibiotics (100 IU of penicillin, 0.1 mg/ml streptomycin, and 5g/ml gentamicin) at 37°C under 5% CO. The medium was then removed, and the parasites were incubated in new medium containing 2% glucose overnight. The supernatant was collected, centrifuged, filter-sterilized (0.2 m), concentrated, and desalted (10 mMTris, NaCl pH7.4) using 3 kDa filters (Centriprep YM-3, Millipore). The protein concentration was determined by the Bradford assay [31].

### Production of anti HcESP polyclonal antibodies (IgG_HcESP_)

To generate polyclonal antibodies against HcESP (**IgG**_**HcESP**_), 0.4 mg of HcESP protein was mixed with Freund’s complete adjuvant (1:1) and injected subcutaneously into 3 female Sprague Dawley (SD) rats [27, 32]. Rats received four doses at 2-week intervals. Ten days after the last injection, the rats were anesthetized with diethyl ether, sera containing specific anti-HcESP antibodies were collected, and the concentration of antibodies was determined by ELISA. The specific reactivity with HcESPs was confirmed by western blot analysis.

### Western blot analysis of the specificity of IgGHcESP

Purified HcESP (20 μg) were resolved by 10% SDS-PAGE and transferred to Hybond-C extra nitrocellulose membranes (Amersham Biosciences, UK). Non-specific binding was blocked by incubating the membranes in 5% skim milk in Tris-buffered saline (TBST) for 1 h at room temperature. The membranes were then washed 5 times (5 min each) with TBS containing 0.1% Tween-20 (TBST), followed by incubation with the primary antibodies (IgG_HcESP_) for 1 h at 37°C (1:100 dilution in TBST). After washing 5 times with TBST, the membranes were incubated with HRP-conjugated rabbit anti-rat IgG (Sigma, USA) for 1 h at 37°C (diluted 1:2000 in TBST). Finally, the immunoreaction was visualized after incubation with freshly prepared diaminobenzidine (DAB, Sigma) as a chromogenic substrate for 5 min.

### Collection of PBMCs from goats experimentally infected with *H*. *contortus*

To identify HcESPPBMC-interacting proteins, three male Boer goats (2 years old) were raised under nematode-free conditions for the *in vivo* experiment. Infective stage larvae (L_3_) of *H*. *contortus* were produced *in vitro*, and 8000 L_3_ were administered to the nematode-free goats. The goats were monitored during the entire experimental period. To confirm *H*. *contortus* infection, fecal samples were collected from the rectum of each infected goat twice each week and checked for the presence of *H*. *contortus* eggs. Food and water were provided to all animals ad libitum. Twenty milliliters of heparinized blood was collected from each goat after 7 (L_4_ stage), 15 (L_5_ stage), 40 (early adult stage) and 60 days (late adult stage) by vein puncture. PBMCs were separated by the standard Ficoll-Hypaque (GE Healthcare, USA) gradient centrifugation method [33], and isolated PBMCs were used to identify HcESP/PBMC-interacting proteins by co-immunoprecipitation (Co-IP), western blot and liquid chromatography–tandem mass spectrometry (LC-MS/MS) analyses.

### Co-immunoprecipitation of HcESPPBMC-interacting proteins

Co-IP was performed using the Protein A/G PLUS-Agarose Immunoprecipitation Kit (Santa Cruz Biotechnology, USA) according to the manufacturer’s instructions. Briefly, 4× 10^7^ PBMCs were collected from experimentally infected goats (*in vivo*) were pelleted and lysed with 3 mL of NP-40 lysis buffer (50 mM Tris pH 7.4, 150mM NaCl, 1% NP-40) containing protease inhibitor cocktail (Merck, USA). Cellular debris was pelleted by centrifugation at 10,000 x g for 10 min at 4°C, and the supernatant was transferred to a new tube. The cell lysate was precleared by incubation with 1 μg of rat normal IgG and 20 μL of Protein A/G PLUS-Agarose beads at 4°C for 30 min. After pelleting the beads by centrifugation at 1,000 ×g for 5 min at 4°C, the protein concentration of the supernatant (cell lysate for IP) was determined using the Pierce^TM^ BCA^TM^ Protein Assay (Thermo Fisher Scientific, USA).

A 1-mL aliquot of the above lysate was incubated with IgG_HcESP_ overnight at 4°C. Immune complexes were isolated using 20 μL of protein A/G plus agarose. Immunoprecipitates were collected by centrifugation at 2,500 rpm for 5 min at 4°C. The supernatant was carefully aspirated and discarded, and the pellet was washed four times with RIPA buffer. After the final wash, the pellet was resuspended in 1X SDS buffer.

### Confirmation of the proteins of HcESP interacted with PBMCs *in vivo* by Western blot

The immunoprecipitates obtained by Co-IP were used to confirm HcESP interation *in vivo* by western blot using IgG_HcESP_ as the primary antibody as described in the previous section_._

### In-solution trypsin digestion and liquid chromatography–tandem mass spectrometry (LC-MS/MS)

In-solution trypsin digestion and LC-MS/MS of immunoprecipitates were performed at Shanghai Applied Protein Technology, Co. Ltd. MS data for protein identification were obtained using Q Exactive (ThermoFinnigan, San Jose, CA). Approximately 30μg of sample was boiled with 30μL of STD buffer in a water bath for 5 min and cooled to room temperature. A 200-μL aliquot of UA buffer (8 M Urea, 150mMTris-HCl, pH 8.5) was added, followed by 30 kDa ultrafiltration centrifugation. After centrifugation, the filtrate was discarded, and 100μL of IAA (50mM IAA in UA) was added. After oscillation for 1 min, the sample was incubated at room temperature in the dark for 30 min, centrifugation was repeated as above, and the filtrate was discarded. Then, 100 μL of UA buffer was added, and the sample was centrifuged twice. Finally, 100μL of 25mM NH_4_HCO_3_ was added and centrifuged twice as described above. The solution was then digested with 40μL of trypsin overnight at 37°C.

Dried peptides were dissolved in 40μL of 0.1% formic acid (FA), and a 20μL aliquot was desalted for 10 min on a C-18 pre-column (Zorbax 300SB-C18 peptide traps, Agilent Technologies, Wilmington) pre-equilibrated with 0.1% FA. Separation was performed by capillary high-performance liquid chromatography (0.15 X 150mm RP C18 analytical column, Column Technology Inc.) at 200°C using a chromatographic gradient of 0.1% FA in H_2_O (A) to 0.1% aqueous FA in 84% ACN (B) over 60 min (liquid linear gradient of solution A: 1–4% (1–50 min), 4 to 50% (50–54 min) and linear gradient of solution B from 50–100% (54–60 min); B was maintained at 100%).

### Database search

Data were searched against an in-house *H*. *contortus* sequence Uniprot database (21,722 protein entries) based on the recently published *Haemonchus* genome [34] using the search engine Mascot (v.2.2, Matrix Science, London, UK), allowing a maximum of two missed cleavages. Carbamidomethyl (C) was specified as a fixed modification and oxidation (M) as a variable modification.

### Gene Ontology (GO)

Gene ontology (GO) annotation was performed using BLAST2GO (version 2.7.2). The sequence alignment software NCBI BLAST + (ncbi-blast-2.2.28 + -win32.ext) was used to compare the identified protein sequences and the protein sequence NCBI nr database. According to the principle of similarity, functional information for homologous proteins can be used for the functional annotation of target proteins. Only results in the top10 and with an E value ≤ 1 e-3 ratio in subsequent sequence analysis were retained. A resulting ratio of similarity of 42–100% was considered.

### Functional annotation improvement by STRING protein clustering analysis

STRING DB (version 9.1) was used to improve the functional annotation and analyze the functional networks among protein families [35, 36]. The sequences of unassigned proteins were retrieved from the UNIPROT-KB and subjected to protein clustering analysis to identify functional protein association networks using the STRING tool (http://string.embl.de/) [37]. Orthologous protein groups matching our queries were used for functional association networks and gene ontology annotation. A STRING conservative score threshold of 0.4 was applied to calculate a confidence score on the basis of the conserved gene neighborhood, gene fusion events, and significant co-occurrence and co expression.

### Validation of proteomic data by interaction analysis of recombinant proteins identified at different developmental stages

To validate the proteomic data, we confirmed the interaction of 6 recombinant proteins identified at different developmental stages to goat PBMCs. The genes encoding 14-3-3 (Hc-ftt), ADP-ribosylation factor (Hc-arf), SCP-like extracellular-domain-containing protein (Hc-scp) and serine threonine kinase (Hc-stp) were cloned using specific reverse and forward primers (Table 1). The genes encoding actin [38], and glyceraldehyde-3-phosphate dehydrogenase (HcGPDH) [39] were previously cloned in our laboratory. Briefly, the ORF of each gene was amplified by RT-PCR and cloned into pMD-19T (Takara Biotechnology). After double digestion with the corresponding restriction enzymes, DNA fragments were recovered and successfully sub-cloned into the pET32a (+) expression vector. *Escherichia coli* BL21 cells containing the recombinant gene expression plasmid were cultured in Luria-Bertani medium with ampicillin (100 μg/mL), and expression of the recombinant proteins (rHc-ftt, rHc-arf, rHc-scp, rHc-stp, rHc-act, rHc-GPDH) was induced by IPTG. The histidine-tagged fusion protein was purified from the bacterial lysates using the His-Bind Resin Chromatography kit (Novagen) and dialyzed in phosphate buffered saline (PBS, pH 7.4) to remove imidazole. The purified recombinant proteins were dissolved in PBS (pH 8.0) containing 0.1mM DTT (PBS/DTT). The purity of the protein preparation was determined by SDS-PAGE. Protein concentrations were determined by the Bradford method. Endotoxins were removed from the recombinant proteins using the ToxinEraser^TM^ Endotoxin Removal kit (GeneScript, USA). Polyclonal antibodies against the recombinant proteins were produced as described above.

**Table 1:** Oligonucleotide primer sequences for PCR.

Name	Accession No.	Sequences(5’- 3’)
*Haemonchus contortus* 14-3-3 (Hc-ftt)	CDJ94531	GGATCCATGGCTGACAATAAGGATG
		GAATTCCAATTTGCACCTTCTCCTT
*Haemonchus contortus*ADP-ribosylation factor (Hc-arf)	CDJ89627	AAAGGATCCATGGGTAACATTTTCGG
		GCGCTCGAGTTATCCTCTGTTTTTCA
*Haemonchus contortus*serine/ threonine protein kinase (Hc-stk)	AF457202	AAGCTTATGGTTCCGGCCTCTTATCAGA
		GAATTCTCGACTGACCGGCAGGAGCTTG
*Haemonchus contortus* SCP-like extracellular-domain-containing protein (Hc-scp)	CDJ81443	GAATTCATGTGTCCAGACACCAATGGTA
		AAGCTTTTATGGGGCAATACAGAGAGCT

### Interaction of recombinant proteins with PBMCs

Heparinized blood was collected by vein puncture from dewormed healthy goats. PBMCs were separated as described in the previous section and washed twice in Ca2+2+/Mg- free PBS pH 7.4. Cell viability assessed by means of the trypan blue exclusion test was consistently >95%. The PBMC were resuspended to a final density of 1×10^5^ cells/ml in RPMI 1640 medium containing 10% heat inactivated fetal calf serum (FCS), 100 U/ml penicillin and 100 mg/ml streptomycin (gibco, Life Technology). PBMCs were incubated in the presence and absence of recombinant proteins (5μg/ml) for 1 h at 37°C. Confirmation of interaction was determined by an immunofluorescence assay (IFA) as described by Yuan et al. [40]. Briefly, washed cells (10^5^ / ml) were fixed with 4% paraformaldehyde on a poly-L-lysine-coated glass slide. The cells were then treated with blocking solution (4% BSA in PBS) for 30 min to minimize background staining. After sequential incubation with rat anti-recombinant protein IgG (1:100) for 2 h and a secondary antibody (1:300) coupled to the fluorescent dye Cy3 (Beyotime, Jiangsu, China), nuclear staining with 2-(4-amidinophenyl)-6-indole carbamidinedihydrochloride (DAPI, 1.5μM; Sigma, MO, USA) was performed for 6 min. Then, protein localization was determined by observing the staining patterns with a 100× oil objective lens on a laser scanning confocal microscope (L SM710, Zeiss, Jena, Germany). Digital images were captured using the Zeiss microscope software package ZEN 2012 (Zeiss, Jena, Germany).

## Results

### Production and analysis of the specificity of anti-HcESP polyclonal antibodies (IgG_HcESP_)

**Fig 1:**
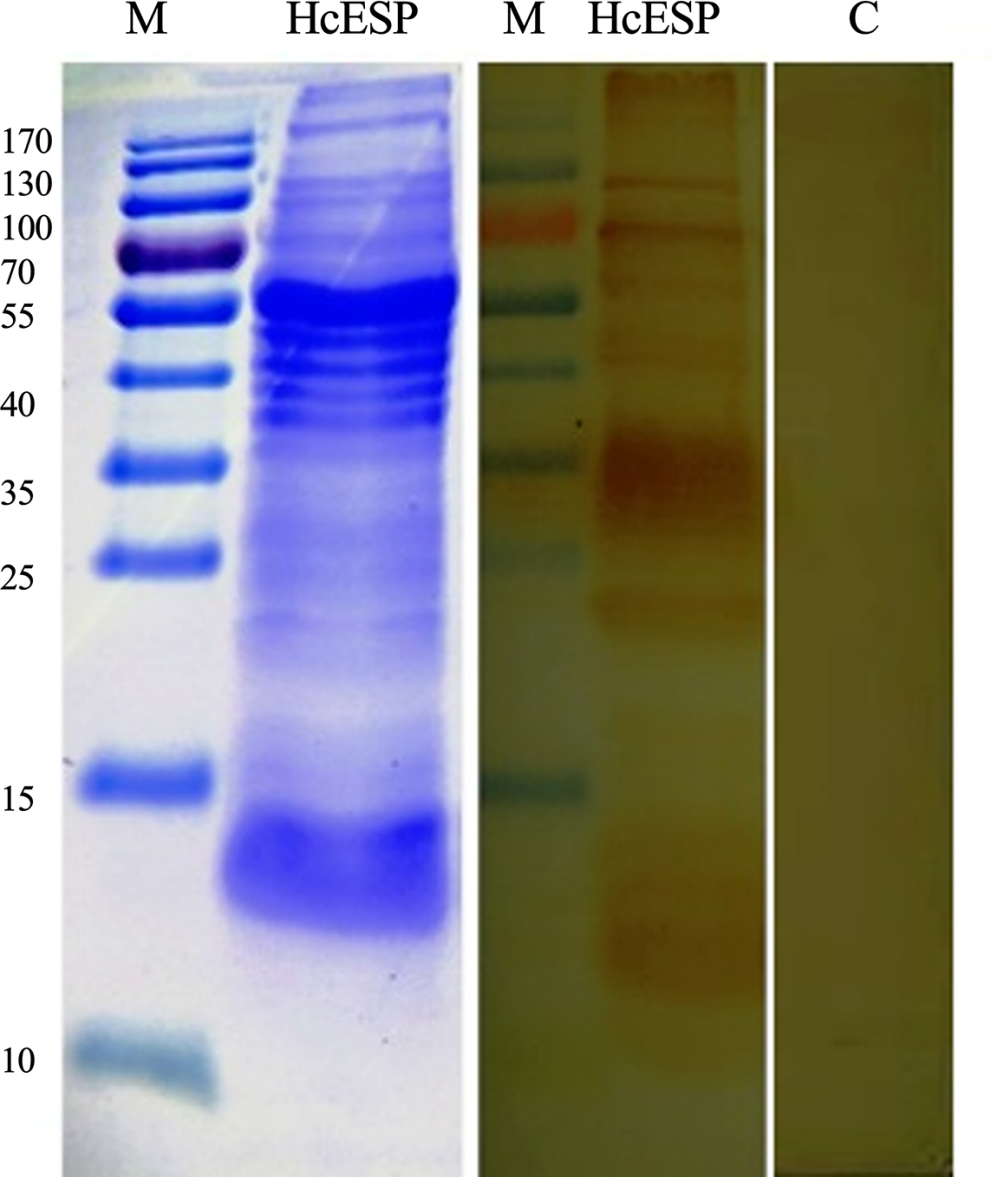
Protein profile of *H*. *contortus* ESP (HcESP) and western blot analysis of HcESP probed with rat anti-HcESP. (c) Control using normal rat serum as the primary antibody.

IgG_HcESP_ was produced by injection of SD rats with HcESP protein mixed with Freund’s complete adjuvant, and the specificity of IgG_HcESP_ was confirmed by western blot using HcESP as the antigen. Normal rat serum was used as a control. Bands from 13 to 180 kDa were detected by IgG_HcESP_, and no bands were recognized by the normal rat serum (Fig 1).

### Confirmation of the interaction of HcESPs with goat PBMCs *in vivo*

**Fig 2:**
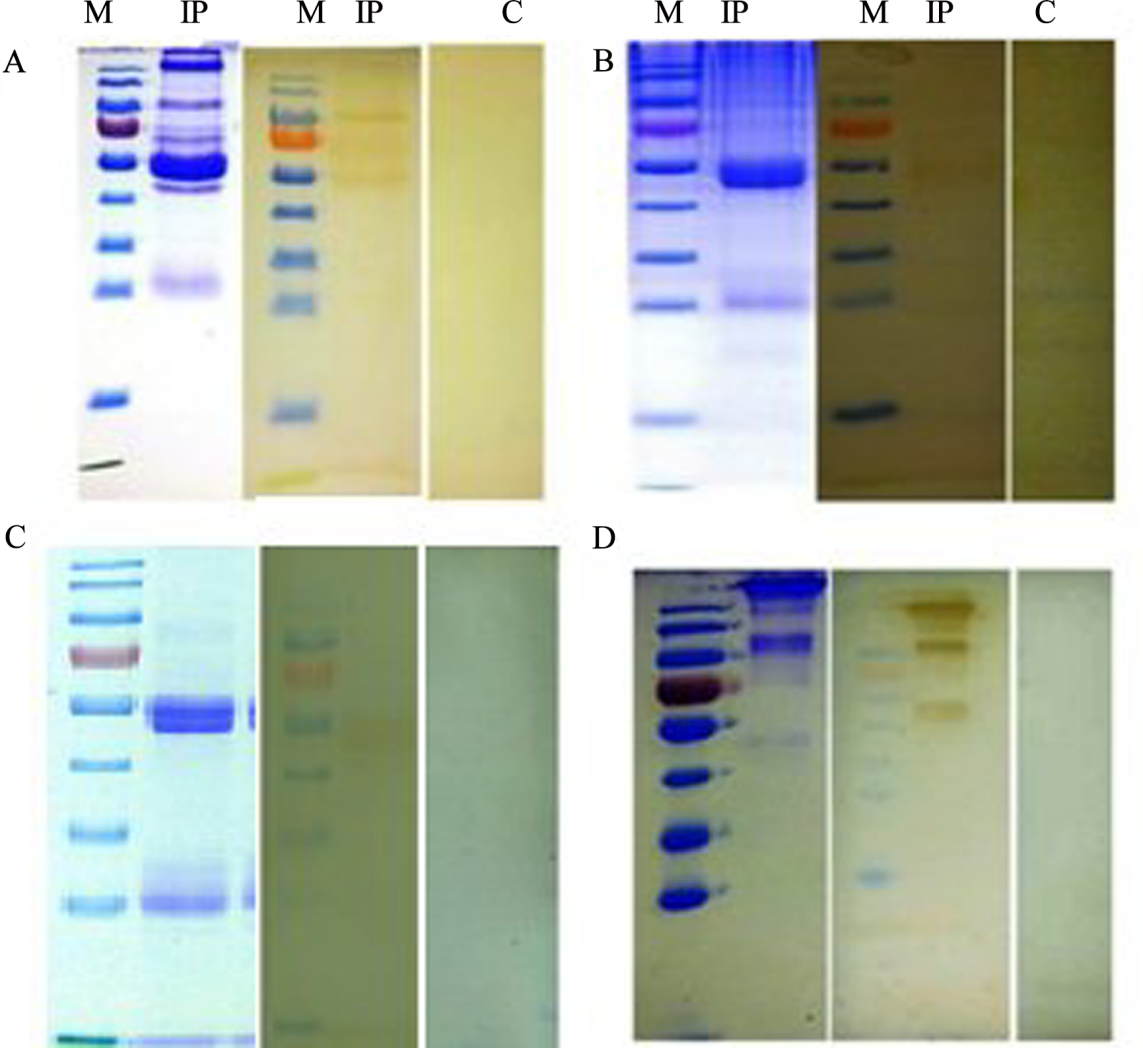
SDS PAGE and western blot analysis of the Co-IP of *in vivo* interacting HcESPs with goat PBMCs at different developmental stages. Marker (M), Immunoprecipitates (IP) and normal rat serum Control (C). (A) L_4_, (B) L5, (C) early adult and (D) late adult.

PBMCs collected from the experimentally infected goats were used to confirm the interaction of HcESP with PBMCs *in vivo*. Protein extracted from the infected goat PBMCs was concentrated by Co-IP. Western blot analysis of the immunoprecipitate using IgG_HcESP_ as the primary antibody confirmed the interaction of HcESPs with the goat PBMCs collected from the experimentally infected goats on day 7 (L_4_ stage), 15 (L_5_ stage), 40 (early adult stage) and 60 (late adult stage) post infection (Fig 2).

### Analysis of HcESP interacting proteins *in vivo* by LC-MS/MS

The interaction of HcESPs with goat PBMCs *in vivo* at different stages of *H*. *contortus* development was analyzed by LC-MS/MS after the interacting proteins were concentrated by Co-IP (S1 Fig). A total of 407 interacting proteins *in vivo* were identified from the *H*. *contortus* protein database via SEQUEST searches. Of these proteins, 47 (11.54%) proteins were common to all developmental stages (S1 Table) including actin, heat shock protein 70, glycoside hydrolase, glyceraldehyde-3-phosphate dehydrogenase, zinc finger, peptidase, Ras domain, serine threonine protein kinase (STK) and 14-3-3.

**Fig 3:**
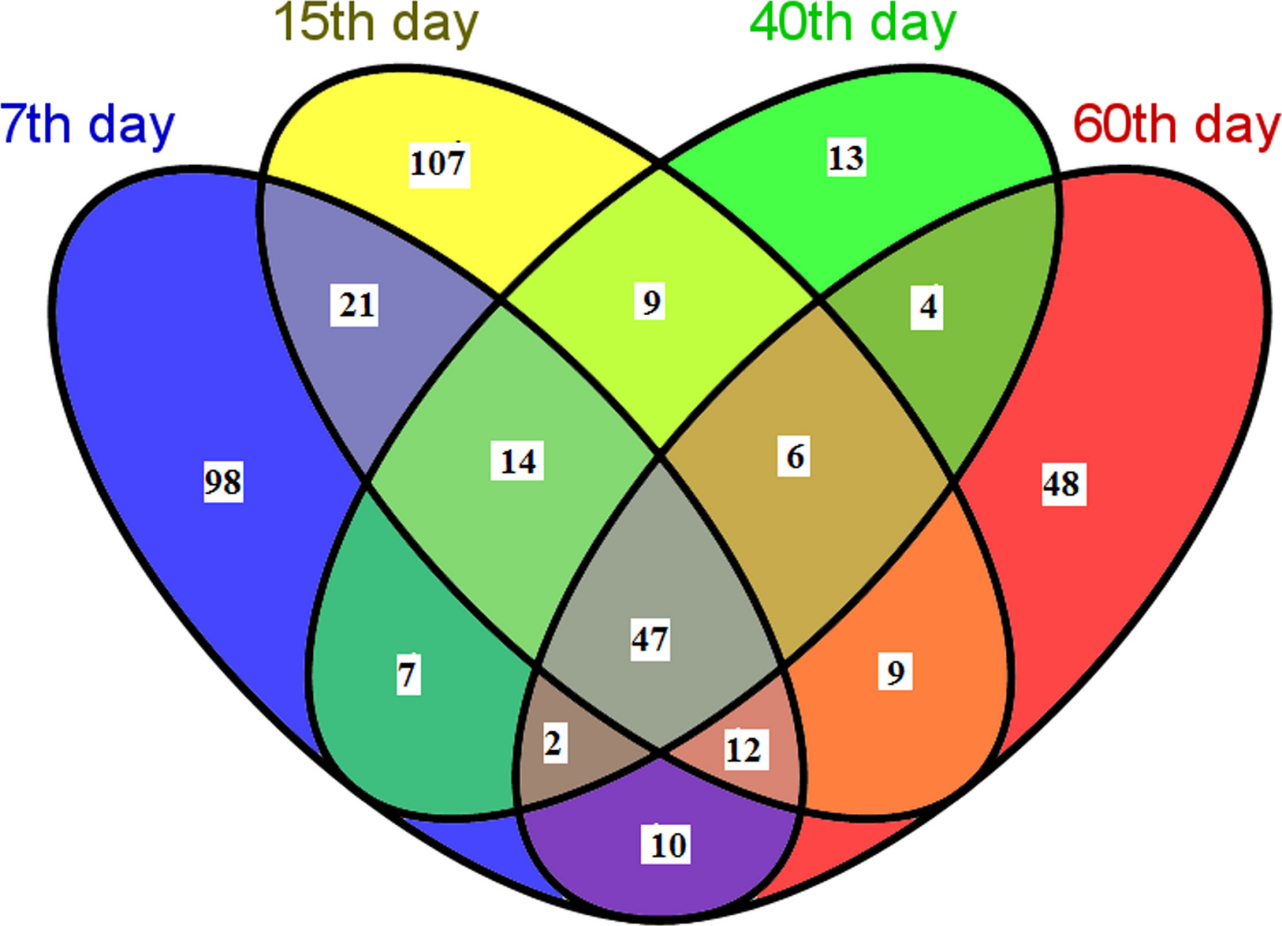
Venn diagram of the interacting proteins shared among different developmental stages *in vivo*.

A total of 94 (23.09%) interacting proteins were common to both the L_4_ and L_5_ developmental stages, including elongation factor 1-alpha, tropomyosin, immunoglobulin I-set and fibronectin, transcription factor E2F dimerization partner (TDP), tenascin-like and cytochrome b5. L_5_ and the early adult stage shared 76 (18.67%) interacting proteins, including major sperm protein (MSP), ribosomal proteins (S8, S5 and L2), Mbt repeat, NADH: ubiquinone oxidoreductase and dynein light intermediate chain. Fifty-nine (14.49%) proteins were shared between the early and late adult stage. The distribution of the interacting proteins at different developmental stages is summarized in S1 Table and Fig 3.

The identified proteins were further categorized according to stage-specific (S2 Table); 98 (24%) interacting proteins were identified at L_4_ including heat shock protein 90 (HSP90), aldehyde dehydrogenase, nematode cuticle collagen, carbohydrate kinase, glucose-methanol-choline oxidoreductase and eukaryotic translation initiation factor 3. At the L_5_ developmental stage, 107 (26.3%) HcESP stage-specific interacting proteins were identified, including enolase, acyltransferase choActase, phosphotyrosyl phosphatase activator, myosin-10, glutamine amidotransferase, annexin, saposin type B and telomerase activating protein Est1. Only 13 interacting proteins were stage-specific in the early adult stage; these proteins included alanine racemase, amino acid transporter domain-containing protein, aminotransferase, and condensation and AMP-dependent synthetase ligase. In the present study, 48 (11.54%) late adult stage-specific proteins were identified, these proteins included CK1/WORM6 protein kinase, protein synthesis factor and translation elongation factor EFTu EF1A and translation elongation factor EFG EF2, selectin-like protein, short-chain dehydrogenase reductase SDR, EVL-14, transcription factor jumonji 1 and tyrosine protein kinase.

### Gene Ontology (GO) analysis

The GO signatures of 234 of the 407 proteins identified *in vivo* were available in the database. To further understand the functions of the proteins identified in this study, we queried the InterPro databases. The identified proteins were classified by molecular function, biological process and cellular component according to the GO hierarchy using a Web Gene Ontology Annotation Plot (WEGO).

**Fig 4:**
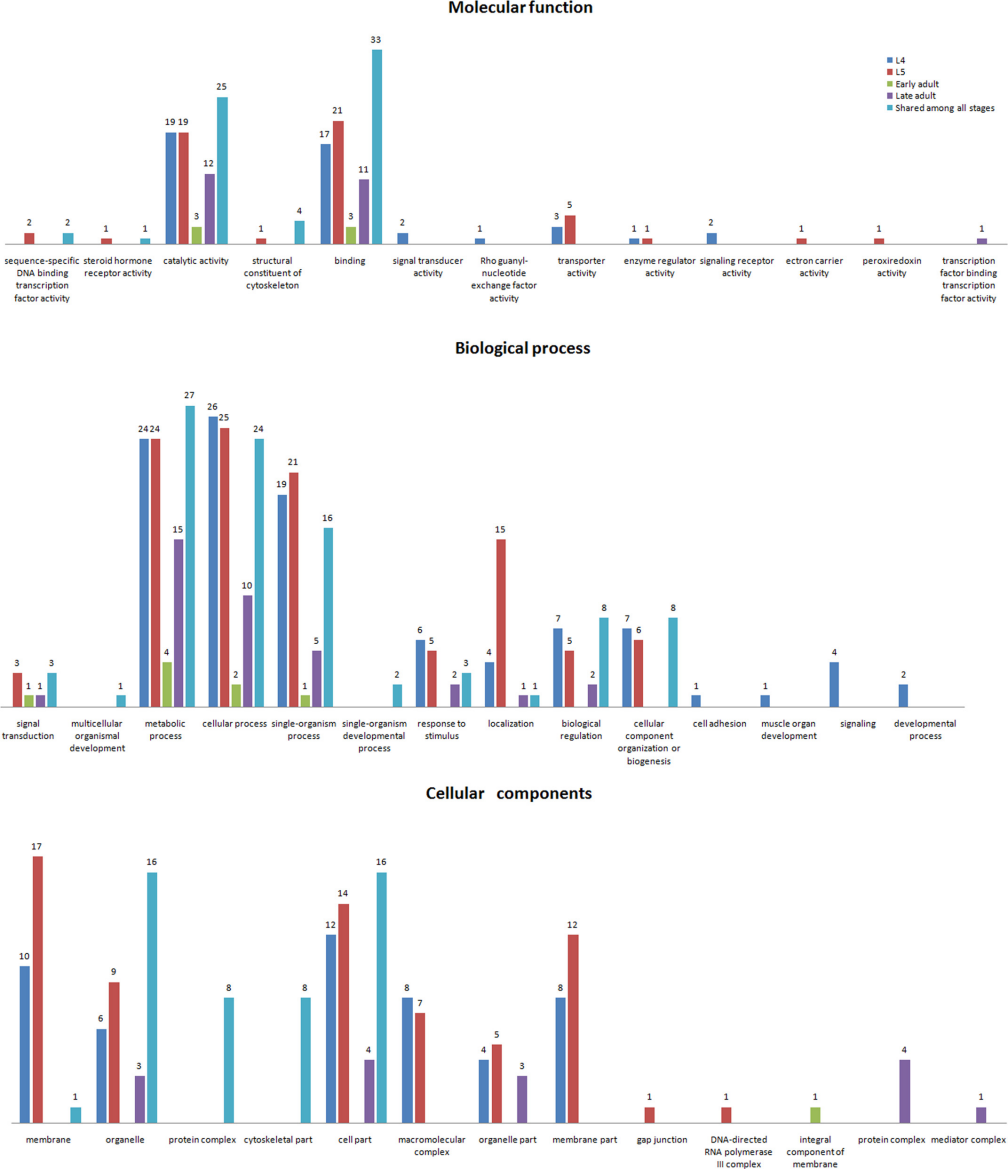
Comparative molecular function, biological process and cellular component GO terms of stage-specific HcESP interacting proteins at different developmental stages and shared among all developmental stages.

Among the 47 shared proteins by all developmental stages, 41 were annotated based on molecular function, and 5 terms were identified. Most were assigned to binding and catalytic activity. For biological processes, 29 proteins were associated with 9 terms. Most of the proteins were related to metabolic, cellular and single organism processes. Among the cellular component annotation, 16 proteins were assigned to 5 cellular component terms, and16 proteins were located in the cell as well as in organelles (Fig 4). GO analysis of the interacting proteins shared between the L_4_ and L_5_ stages resulted in the annotation of 7 molecular functions; most of the proteins were assigned to binding and catalytic activity. For biological processes, 10 terms were identified, primarily metabolic, cellular and single organism processes.

**Fig 5:**
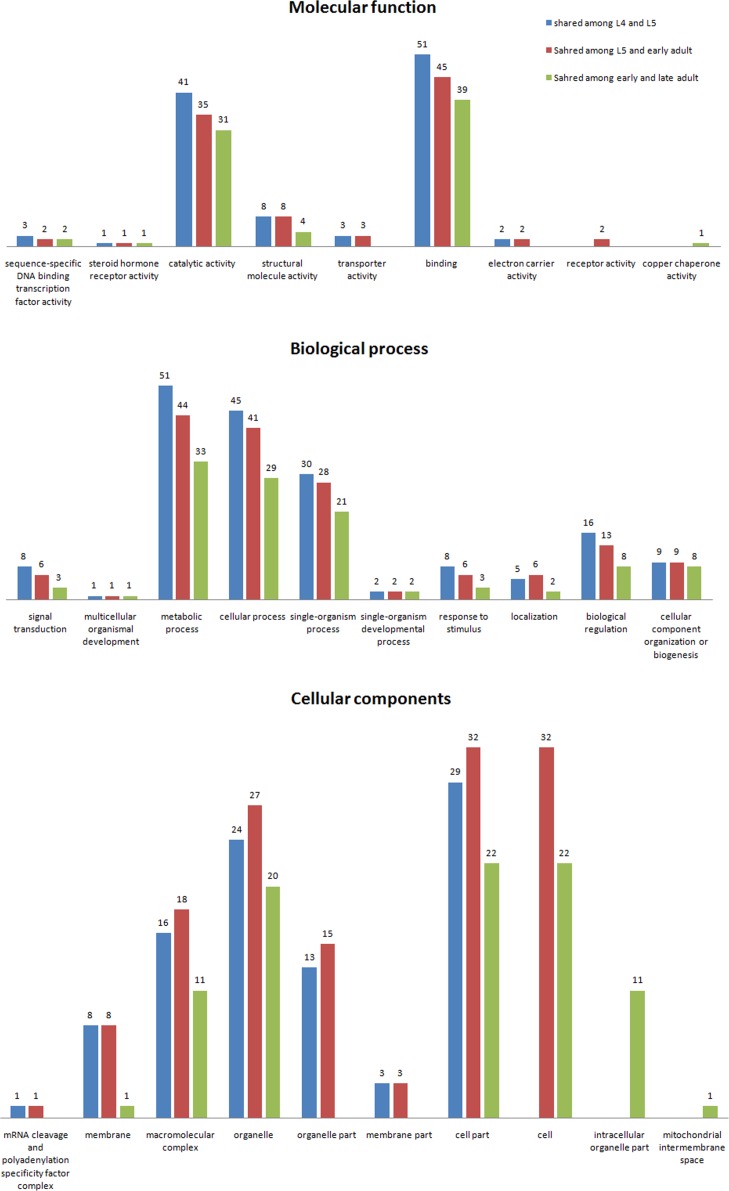
Comparative molecular function, biological process and cellular component GO terms of proteins shared between the L_4_ and L_5_, L_5_ and early adult, and early adult and late adult stages.

An identical distribution of GO terms was observed for the proteins shared between the L_5_ and early adult stages. Eight terms were identified and binding and catalytic activities were the major molecular functions. Among the proteins shared by the early and late adult stages, 39 were assigned to binding and 31 to catalytic activity. The distribution of GO terms was nearly identical for the early and late adult stages (Fig 5).

Among the 98 L_4_ stage-specific proteins, 35 were assigned to 8 molecular function terms. The most abundant terms were catalytic and binding activity. For biological processes, 11 terms were identified. Cellular, metabolic, single-organism and localization were the most abundant terms. According to the cellular component annotation, 7 cellular locations were assigned to 21 proteins. Cell part, membrane, membrane part and macromolecular complexes were annotated as the major cellular components. The results of the GO analysis for the 108 L_5_ stage-specific proteins revealed that 35 proteins were annotated with 9 molecular functions. Binding and catalytic activity were the major terms. For biological processes, 39 proteins were assigned 8 terms; metabolic, cellular process, single-organism process and localization were the most abundant biological processes. In the case of cellular component annotation, 28 proteins were annotated with 8 cellular locations, and most of the annotated proteins were membrane, cell part and membrane part. Among the 13 early adult stage-specific interacting proteins, only 2 terms related to molecular function were annotated, and 3 proteins were assigned to binding and catalytic activity. Metabolic processes were a major biological process at this stage, and only one protein was annotated as a membrane part. GO analysis of 48 late adult stage-specific proteins revealed a molecular function annotation for 18 proteins. The same pattern of functional distribution described above was observed, and 5 GO terms related to molecular function were identified. Catalytic and binding activity were the most abundant terms. Seventeen proteins were annotated as biological process, and 7 terms were attained. Metabolic, cellular and single-organism processes were the major terms. For cellular components, 7 terms were identified for 6 proteins. Protein complex and cell part were highly represented terms (Fig 4).

### Improvement of functional annotation by protein clustering

**Fig 6:**
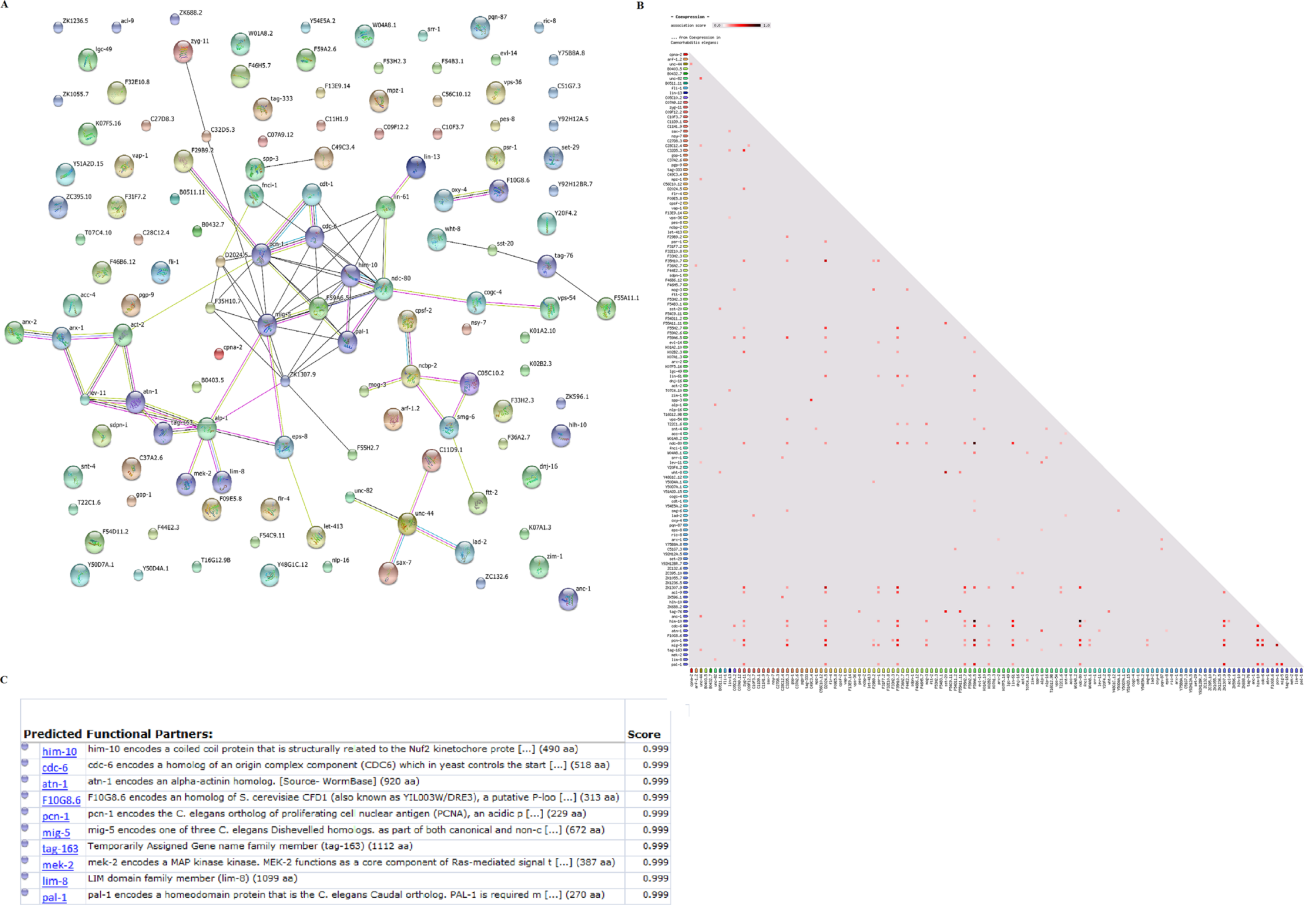
Improvement of functional annotation based on protein clustering. (A) STRING functional protein association network of the predicted associations of unassigned proteins. Nodes of different colors indicate clustering proteins matching our queries. (B) Co-expression graph from *C*. *elegans*. (C) Predicted protein functional partners.

**Fig 7:**
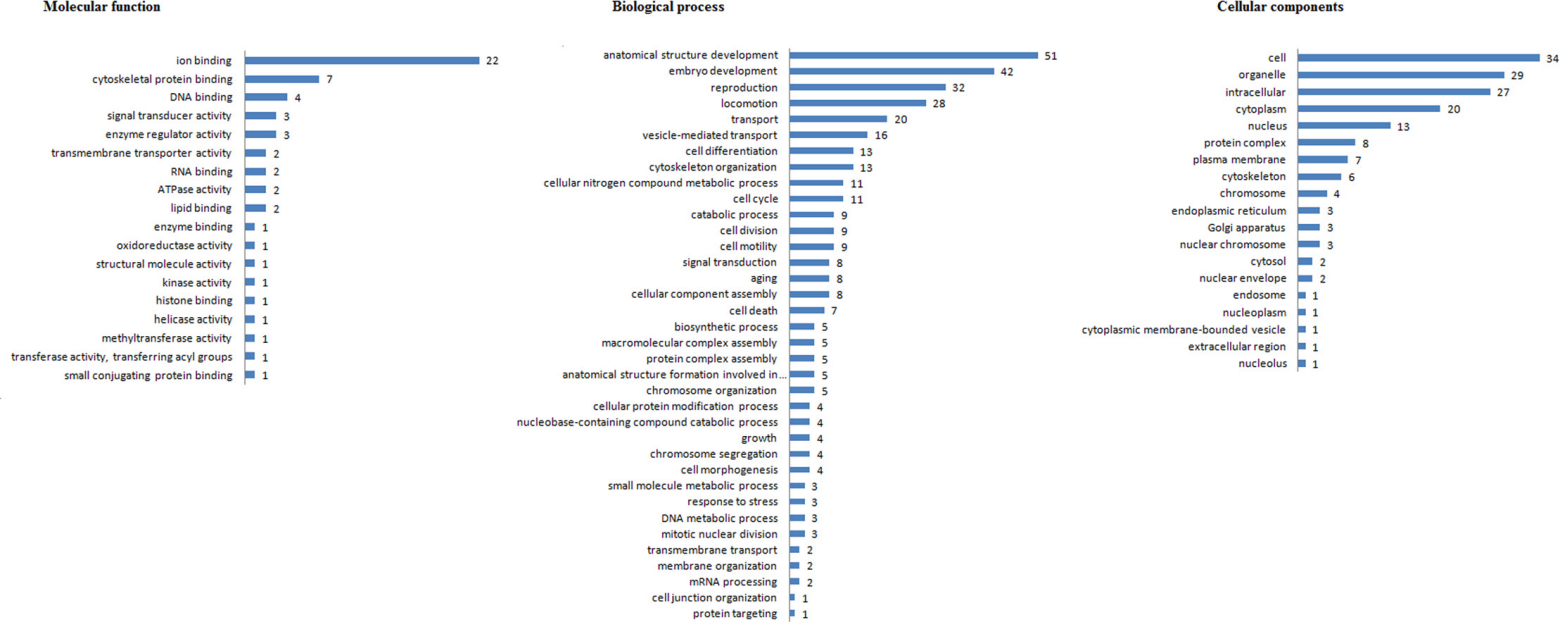
Distribution of Gene Ontology terms improved by protein clustering of 173 unassigned HcESP proteins that bound goat PBMCs at different developmental stages.

The sequences of 173 unassigned interacting proteins were retrieved from UNIPROT-KB and subjected to protein clustering analysis to determine their functional association network in the STRING database. In the STRING database, 118/173 (68.20%) proteins were available, and 80 functional associations were predicted. Fig 6 illustrates the functional interaction between protein orthologues in the nearest organism (*Caenorhabditis elegans*) and their predicted functional partners. The resultant orthologous protein groups were used to predict function. The results of the protein clusters and corresponding functional information are summarized in S3 Table and Fig 7.

Based on the protein clustering, we discovered new HcESP proteins previously annotated as hypothetical proteins in the database. An unnamed protein (U6NP15) identified at the L_4_ and L_5_ stages matched WHiTe (Drosophila)-related ABC transporter family member (wht-8) with 56% similarity. An unnamed protein (W6NHX8) identified at the L_4_ stage matched the P-glycoprotein subclass of the ATP-binding cassette (ABC) transporter super family with 66% homology. ADP-ribosylation factor (arf-1.2) family matched hypothetical protein (U6PBJ7). The identified protein had 97% similarity with C. *elegans*.

Protein cluster analysis enabled the functional characterization of 42 additional proteins based on GO analysis of homologous proteins. Binding activity was a highly represented molecular function including ion binding, cytoskeleton protein binding and DNA binding. Thirty-six biological process terms were identified for 56 proteins. Anatomical structure development, embryo development, reproduction, locomotion and transport were highly represented terms.

### Validation of proteomic data by assessing recombinant HcESPs interacted with PBMCs

**Fig 8:**
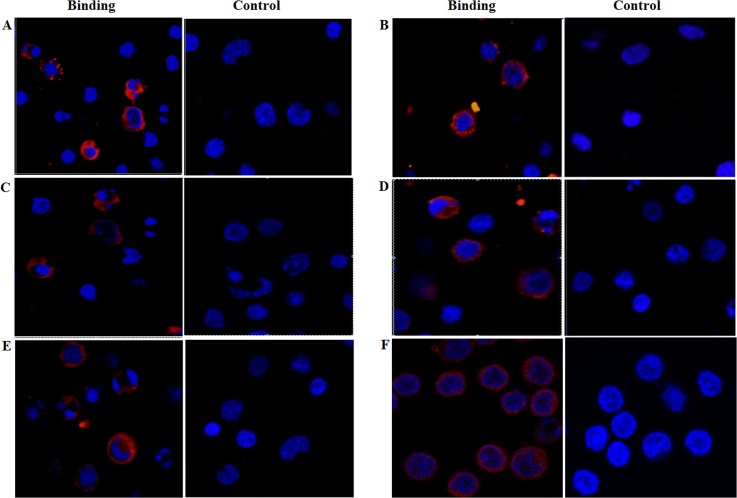
Interaction of recombinant proteins **(A):** rHc-ftt, (**B):** rHc-arf, (**C):** rHc-scp, **(D):** rHc-stp, **(E):** rHc-act and **(F):** rHcGPDH) to PBMC. PBMCs were untreated as controls or treated with the recombinant proteins. The cell nuclei were visualized by DAPI (blue) staining. Staining of the target proteins (red) was visualized by a Cy3-conjugated secondary antibody.

To validate the proteomic data, we purified 6 recombinant proteins (rHc-ftt, rHc-arf, rHc-scp, rHc-stp, rHc-act and rHcGPDH) identified at different developmental stages as interacting proteins with goat PBMCs. Interaction of the recombinant proteins with goat PBMCs was confirmed by immunofluorescence. Nuclei were stained with DAPI (blue fluorescence), and confocal microscopy images revealed that the recombinant proteins were interacted with the cell surface (red fluorescence). In the control group, no red fluorescence was observed (Fig 8).

## Discussion

Excretory and secretory products (ESPs) are produced and released by parasites during *in vitro* cultivation [11] and *in vivo* [12]. *H*. *contortus* excretory and secretory products (HcESPs) contain many proteins [16] and performed diverse functions such as tissue penetration and host protein degradation [14]. It was previously reported that early expression of T_H_2 cytokine IL-4 and upregulation of genes that recruit neutrophils (CXCL1) and macrophages (MCP1) was associated with the resistance to *H*. *contortus* [41]. Estrada-Reyes et al. [42] reported the high regulation of IL-5 at 2, 7 and 14 days post-infection (PI) and IL-6 at 14 days PI of *H*. *contortus*. In our previous study, HcESPs displayed suppressive potential on the goat PBMCs *in vitro*. They inhibited the productions of IL-4, IFN-*γ*, nitric oxide, cell proliferation, whereas increased the production of suppressive cytokine IL-10, inflammatory modulator IL-17 and cell migration [30]. However, the protein or proteins of HcESPs that govern the functions of HcESPs *in vitro* or *in vivo* are unknown. In the present study, the interactions of HcESP at different developmental stages with goat PBMCs *in vivo* were evaluated by Co-IP followed by LC-MS/MS. A total 407 non-redundant *H*. *contortus* proteins that interacted with goat PBMCs *in vivo* were identified by searching the *H*. *contortus* Uniprot database. This study is the first to report the *in vivo* identification of HcESP interacting proteins from the L_4_ to adult stage of worms living in the goat host. We purified 6 recombinant HcESP interacting proteins to validate the proteomic data and the interaction of these proteins with goat PBMCs by IFA.

In our study, 47 HcESP interacting proteins were common among all developmental stages. Among them, 4 peptidase proteins including cysteine peptidase (C46), serine peptidases (S9 & S28) and metalloprotease (M13) were identified. Significant expression of all three classes of peptidases in the parasitic L_4_ to adult stages has been reported [43]. Schwarz et al. reported more than 120 upregulated peptidase genes in *H*. *contortus* parasitic stages, and various clans were predicted to be secreted peptidases, including metallopeptidases (M 12A, M01, M13, M12A, M10A), aspartic peptidases (A01A) and cysteine peptidases (CA01A) [10]. The identification of secreted proteins related to the peptidase family in the current research supports these reports, which indicated that these proteins play a crucial part in the catabolism of globin by hemoglobin cleavage [1]. Development from L_3_ to L_4_ *in vitro* leads to the release of a metalloprotease enzyme that inhibits blood clotting and thus facilitates blood feeding [1, 44]. We confirmed the above finding *in vivo* and report for the first time the presence of these peptidase proteins in the blood circulation. These findings support previous genomic studies reporting that genes encoding peptidase proteins are transcribed at a higher level in the host compared to free-living stages [10, 16].

In the present study, a key glycolytic enzyme, glyceraldehyde-3-phosphate dehydrogenase (GPDH), was identified as interacting protein with goat PBMCs *in vivo* in all developmental stages. GPDH plays an important role in host invasion of the worm in addition to its glycolytic activity [45, 46]. This protein could play a key role in immune modulation by binding complement (C3) and thus inhibiting complement activity [2]. GPDH is essential for inducing the T helper (Th1 and Th17) immune response during natural infection [47]. Our findings suggested that GPDH plays an important role in immune modulation and is one of the most important HcESP proteins.

In the present study, the serine/threonine kinases (PKs) were also identified in all stages. This finding is in agreement with the results of previous studies [10, 48]. PKs play a vital role in the cellular signaling transduction involved in cell proliferation, differentiation, cell-cycle progression, transcription, DNA replication, metabolic processes, phosphorylation, apoptosis, autophagy and inflammation [49–52]. The interaction of *H*. *contortus* PKs to goat PBMCs suggests that this kinase might also play a significant role in the functional regulation of goat PBMCs.

We identified the secreted *H*. *contortus* 14-3-3 protein as a goat PBMC-interacting protein in all parasitic stages. Gene sequences of 14-3-3 have been reported for several protozoan and metazoan parasites, including *Plasmodium falciparum*, *Trypanosoma cruzi*, *Toxoplasma gondii*, *Neosporacaninum*, *Eimeria tenella*, *Schistosoma japonicum*, *Echinococcus granulosus*, *Meloidogyne incognita* [53–59]. 14-3-3 proteins are phosphoserine-binding proteins that control the actions of a wide range of targets via direct protein–protein interactions. In animal cells, the majority of the known targets of 14-3-3 proteins are involved in signal transduction, transcription and proliferation [60–62]. Our findings indicate that the *H*. *contortus* 14-3-3 protein may act in signal transduction.

The cytoskeletal protein actin was identified as an interacting protein in all stages. Actin has been detected in different helminths [63, 64] and is involved in very important cellular functions, including cell division, secretion, signaling, cellular shape and volume regulation, movement and phagocytosis [65, 66]. The effects of the interaction of *H*. *contortus* actin with host PBMCs merits further study.

We observed that *H*. *contortus* HSP70 interacted with goat PBMCs *in vivo* in all developmental stages. HSP70 proteins are molecular chaperones that play important roles in the process of invasion, response to stress and survival in nematodes. HSP70 has been identified in several parasitic nematodes [67–69], but there is minimal information available for *H*. *contortus* [70, 71].

The transition from the L_3_ to L_4_ stage is key to the establishment of parasitism by *H*. *contortus*. ESPs play a very important role in pathogenesis and induce immune modulation at the early stage of infection. L_4_ is the first blood-feeding stage of *H*. *contortus*, and at this stage, genes related to motor activity and metabolism occur in the parasite [72]. Here, we observed high proteomic complexity of the HcESPs that interacted with goat PBMCs at different developmental stages. HcESP interacting proteins were more abundant at the L_4_ and L_5_ stages than at the early and late adult stages [73]. A previous study reported that 234 proteins were upregulated in the L_4_ stage compared to L_3_ [10]. In our study, 209 and 217 proteins were identified at the L_4_ and L_5_ stages, respectively, and 94 interacting proteins were shared between the 2 stages. Most of the shared proteins were related to binding (n = 51), catalytic activity (n = 41) and metabolism (n = 51).Our findings suggest the active involvement of these proteins in parasitism and immunemodulation.

SCP-like extracellular-domain-containing protein (vap-1) was identified as an interacting protein in the L_4_ and L_5_ stages. Vap-1 encodes a predicted secreted protein that is similar to the venom allergen-like proteins reported in a number of invertebrates, including parasitic nematodes [74–76]. Schwarz et al. identified 82 genes related to SCP proteins including 54 upregulated genes in the parasitic stages [10]. Previously, two proteins related to the SCP-like proteins Hc24 and Hc40 were reported in the ESPs of adult *H*. *contortus* [12, 77]. Our findings support these previous results, and the interaction of these proteins with goat PBMCs at multiple stages *in vivo* suggests a critical role of SCP-like proteins in infection and may be immunomodulatory factors.

Elongation factor-1α protein is involved in signaling activity and was identified in our study at L_4_ and L_5_. EF-1α is highly conserved and ubiquitously expressed in all eukaryotic cells [78]. EF-1α proteins has been reported in parasites including *Cryptosporidium hominis, Trichomonas vaginalis, Trypanosoma brucei, Clonorchis sinensis* and *Brugia malayi* [79–83] Functionally, EF-1α transfers aminoacylated tRNAs to the ribosome A site in a GTP-dependent reaction [84]. In addition, EF-1α appears to have a number of other functions associated with cell growth, motility, protein turnover, and signal transduction [85, 86], DNA replication/repair protein networks [87] and apoptosis [88]. The interaction of EF-1α with host PBMCs indicates its active role as an immune depressant and warrants further investigation.

A total of 102 proteins were identified at the early adult stage, and 76 were shared between the L_5_ and early adult stages. Fewer HcESPs interacted at the adult stage compared to the L_4_ and L_5_ stages. Thus, the parasite releases more ESPs in the early stages of infection to modulate immune function for parasite survival. Studies of expressed sequence tag (EST) data have provided transcriptional and genomic insights on the different developmental stages of *H*. *contortus* [10, 59, 72, 89]. We identified 3 ribosomal proteins (S8, S5 and L2) at the L_5_ and early adult stages. Zamanian et al. [83] identified 14% ribosomal proteins of the *B*. *malayi* exosome-like vesicles (ELVs) released from the infective L3 stage. Ribosomal proteins actively participate in cellular processes other than protein biosynthesis and can act as components of the translation apparatus, cell proliferation and apoptosis [90]. Cantacessi et al. employed an *in silico* subtraction approach to identify *H*. *contortus* L_3_ and xL_3_ genes and predicted that *H*. *contortus* L_3_-specific genes encoding ribosomal proteins [91] were required for phagocytosis [91, 92]. Our findings confirm these previous reports, and the participation of ribosomal proteins in cellular processes should be further investigated.

We identified 137 proteins at the late adult stage. Fifty-nine proteins were shared between the early and late stages. Among them, serine/threonine-protein phosphatase (STPs) was identified as an important interacting protein. STPs from various parasites have been functionally characterized [93–95]. Protein phosphatases are involved in major biological processes such as cell division, apoptosis and exocytosis [96]. STPs are often involved in signal transduction and transcriptional activation [97–99]. Our findings suggest that *H*. *contortus* STPs might be involved in various biological processes, particularly signal transduction.

We identified 98 stage-specific HcESP interacting proteins in the L_4_ developmental stage, including heat shock protein 90, extracellular ligand-binding receptor, aldehyde dehydrogenase, carbohydrate kinase, myosin-4, aldehyde dehydrogenase and glucose-methanol-choline oxidoreductase. A total of 107 L_5_ stage-specific proteins were identified, including enolase, saposin type B, myosin-10, aromatic amino acid beta-eliminating lyase threonine aldolase and annexin. Genes or ESTs transcribed during different developmental stages have been investigated previously [10, 72]. Hartman et al. reported that the cysteine protease, Hc42, Hc60 and vitellogenin genes were transcribed at the adult stage, whereas glutathione peroxidase, alpha-tubulin, Hc43 and Hc38 were transcribed at the L_3_ and adult stages [44]. The immunological involvement of these stage-specific HcESP proteins remains to be further characterized.

Scaffold proteins are essential components of signaling functions such as the trafficking, anchoring and multimerization of glutamate receptors and act as adhesion molecules [100–102]. We identified 53 HcESP scaffold proteins that interacted with goat PBMCs at different developmental stages *in vivo*, including 27 in L_4_ and 28 in L_5_. Interestingly, only 5 and 10 scaffold proteins were observed at the early and late adult stages. Low concentrations of scaffold proteins increase the output of cascades, but as the concentration of the scaffold proteins increases, the output of the cascade decreases. At an elevated concentration of scaffold proteins, one molecule can bind only one kinase molecule, and thus the output of the signaling cascade is also very low [103, 104]. The high concentration of scaffold proteins observed at the L_4_ and L_5_ stages in the present study could represent a mechanism of immune modulation by combinatorial inhibition of the signaling cascade.

We identified the hypothetical *H*. *contortus* protein (U6PBJ7) as a member of the ADP-ribosylation factor (arf-1.2) family by STRING protein clustering. This highly conserved family is involved in a wide range of cell functions. ARF proteins are N-myristoylated GTPases, which are involved in membrane trafficking, actin cytoskeleton, regulation of apoptotic fate and activation of phospholipase D1 (PLD1) and phosphatidylinositol 4-phosphate 5-kinase [105–107]. The present study is the first to report interaction of the HcESPARF-1.2 protein with goat PBMCs *in vivo* at multiple developmental stages. In our study, another hypothetical HcESP protein (W6NHX8) identified at the L_5_ stage was confirmed as P-glycoprotein-9 (pgp-9), part of the ATP-binding cassette (ABC) or traffic ATPase subclass, by STRING protein clustering analysis. The *H*. *contortus* Pgp gene may be involved in host-parasite interaction, particularly in eosinophil granule product detoxification [108, 109]. Nematode parasites undergo important adaptations during the transition from free-living to parasitic stages, such as evasion of the host immune reaction, metabolism and growth. Issouf et al. [108] compared the expression level of Pgps in free and parasitic stages and reported that Hco-pgp-9.2, Hco-pgp-11, Hco-pgp-3 and Hco-pgp16 mRNAs were over expressed in the L_4_ and adult stages. Here we report for the first time that *H*. *Contortus* P-glycoprotein-9 (pgp-9) interacted with goat PBMCs at the L_5_ stage *in vivo*.

Secreted extracellular vesicles (EVs) play an important role in parasite-host interactions. Exosomes considered highly bioactive EVs that facilitated cell to cell communication in many eukaryotes and prokaryotes [110]. Several studies on various parasites including helminths demonstrated that EVs could carry and deliver virulence factors such as proteins and sRNAs to the host [111–115]. In the present study, various HcESPs interacting proteins including annexins, GAPDH, actin, HSP70, HSP90, 14-3-3 proteins, tubulin, ras-related protein, histone, ATP synthase subunit alpha, HSP DnaJ, eukaryotic translation initiation factor 3, enolase, ribosomal proteins and acyltransferase ChoActase were identified at various developmental stages *in vivo*. In the previous studies these interacting proteins were recognized as members of EVs [83, 116, 117]. The interaction of these EVs related HcESPs with goat PBMCs suggested their important regulatory role in host–parasite interaction.

To provide a comprehensive understanding of the roles of the *H*. *contortus* proteins that interact with host PBMCs, the identified proteins were functionally categorized based on the GO annotation of molecular functions, biological processes and cellular components. Of the 407 proteins identified *in vivo*, 173 (42.50%) did not have assigned GO terms. These unannotated proteins were further analyzed by clustering of homologous proteins via STRING databases to enhance the functional annotation prediction.

For molecular function GO annotation, the most enriched functions of the HcESPs were related to binding activity. Proteins associated with these functions are involved in ATP binding, nucleotide binding, protein binding, GTP binding, DNA binding, motor activity, translation elongation factor activity, GTPase activity, protein kinase C inhibitor activity, protein hetero- and homodimerization activity, protein polymerization and signaling activity [16, 118, 119]. The biological process GO results revealed that the most represented categories were annotated as transport, metabolic, catabolic and phosphorylation processes. A nearly identical profile of biological process annotation was reported by Moreno et al. for *Heligmosomoides polygyrus* ESPs [118].

The functional annotation of proteins can be predicted and improved by clustering of homologous proteins. The functional annotation of parasite proteins is often constrained by the small proportion of genes with homologs in model organisms [120]. However, based on the clustering of homologous proteins [50, 121], we were able to enrich the GO annotations of 173 unannotated proteins identified at different developmental stages. Clustering the homologous proteins increased the GO annotation by 24.27% for molecular function annotation, 23.36% for biological process and 22.54% for cellular components.

In conclusion, we analyzed the interaction of *HcESP*s with host PBMCs *in vivo* at different developmental stages. Many of the identified proteins were highly developmentally specific proteins. The large numbers and the complexity of the interacting proteins indicated that the HcESPs interact with the host immune cells in complex ways and result in complex regulation of the host immune cells. Our data provide a baseline for understanding the relationship between the parasite and host. However, the interacting partners and the regulatory mechanisms of specific proteins remain to be further investigated. The functions of the novel interacting proteins and the nature of the unassigned proteins also require further study.

## Supporting Information

S1 Fig Digestion of peptides by trypsin and MS identification using capillary high-performance liquid chromatography. Total ion flow diagram of HcESPs interacted with goat PBMCs at the (A) L_4_, (B) L_5_, (C) early adult and (D) late adult stages.
(TIF)

S1 Table List of identified interacting proteins of HcESP with goat PBMCs shared among different developmental stages *in vivo*. (DOCX)

S2 Table List of developmental stage specific interacting proteins of HcESP with goat PBMCs identified at different time points *in vivo*. (DOCX)

S3 Table List of proteins available on STRING database matched with our Query sequences of unassigned proteins analyzed for functional annotation improvement by protein clustering analysis. (DOCX)

## References

[1] Nikolaou S, Gasser RB. Prospects for exploring molecular developmental processes in Haemonchus contortus. International journal for parasitology. 2006;36(8):859–68. Epub 2006/06/09. 10.1016/j.ijpara.2006.04.007 . 16759659

[2] Sahoo S, Murugavel S, Devi IK, Vedamurthy GV, Gupta SC, Singh BP, et al Glyceraldehyde-3-phosphate dehydrogenase of the parasitic nematode Haemonchus contortus binds to complement C3 and inhibits its activity. Parasite immunology. 2013;35(12):457–67. Epub 2013/08/10. 10.1111/pim.12058 . 23927077

[3] Blackburn HD, Rocha JL, Figueiredo EP, Berne ME, Vieira LS, Cavalcante AR, et al Interaction of parasitism and nutrition in goats: effects on haematological parameters, correlations, and other statistical associations. Veterinary parasitology. 1992;44(3–4):183–97. Epub 1992/10/01. . 1466129 10.1016/0304-4017(92)90116-q

[4] Jasmer DP, Lahmers KK, Brown WC. Haemonchus contortus intestine: a prominent source of mucosal antigens. Parasite immunology. 2007;29(3):139–51. Epub 2007/02/03. 10.1111/j.1365-3024.2006.00928.x . 17266741

[5] Gilleard JS. Understanding anthelmintic resistance: the need for genomics and genetics. Int J Parasitol. 2006;36(12):1227–39. Epub 2006/08/08. S0020-7519(06)00240-2 [pii] 10.1016/j.ijpara.2006.06.010 . 16889782

[6] Redman E, Sargison N, Whitelaw F, Jackson F, Morrison A, Bartley DJ, et al Introgression of ivermectin resistance genes into a susceptible Haemonchus contortus strain by multiple backcrossing. PLoS Pathog. 2012;8(2):e1002534 Epub 2012/02/24. 10.1371/journal.ppat.1002534 22359506 PMC3280990

[7] Gilleard JS. Haemonchus contortus as a paradigm and model to study anthelmintic drug resistance. Parasitology. 2013;140(12):1506–22. Epub 2013/09/04. 10.1017/s0031182013001145 . 23998513

[8] Kaminsky R, Ducray P, Jung M, Clover R, Rufener L, Bouvier J, et al A new class of anthelmintics effective against drug-resistant nematodes. Nature. 2008;452(7184):176–80. Epub 2008/03/14. 10.1038/nature06722 . 18337814

[9] Stoll NR. Studies with the strongyloid nematode, Haemonchus contortus. Am J Hyg. 1929;10:384–418.

[10] Schwarz EM, Korhonen PK, Campbell BE, Young ND, Jex AR, Jabbar A, et al The genome and developmental transcriptome of the strongylid nematode Haemonchus contortus. Genome Biology. 2013;14(8):R89–R. 10.1186/gb-2013-14-8-r89 PMC4053716. 23985341 PMC4053716

[11] Rathore DK, Suchitra S, Saini M, Singh BP, Joshi P. Identification of a 66 kDa Haemonchus contortus excretory/secretory antigen that inhibits host monocytes. Veterinary parasitology. 2006;138(3–4):291–300. Epub 2006/03/07. 10.1016/j.vetpar.2006.01.055 . 16517075

[12] Schallig HD, van Leeuwen MA, Cornelissen AW. Protective immunity induced by vaccination with two Haemonchus contortus excretory secretory proteins in sheep. Parasite immunology. 1997;19(10):447–53. Epub 1998/01/07. . 9372512 10.1046/j.1365-3024.1997.d01-148.x

[13] Knox DP. Development of vaccines against gastrointestinal nematodes. Parasitology. 2000;120 Suppl:S43–61. Epub 2000/06/30. . 10874709 10.1017/s0031182099005764

[14] Cox GN, Pratt D, Hageman R, Boisvenue RJ. Molecular cloning and primary sequence of a cysteine protease expressed by Haemonchus contortus adult worms. Mol Biochem Parasitol. 1990;41(1):25–34. Epub 1990/06/01. . 2385265 10.1016/0166-6851(90)90093-2

[15] Sun Y, Yan R, Muleke CI, Zhao G, Xu l, Li X. Recombinant galectins of Haemonchus contortus parasite induces apoptosis in the peripheral blood lymphocytes of goat. Int J Pept Res Ther. 2006;13(3):387–92. 10.1007/s10989-006-9045-0

[16] Yatsuda AP, Krijgsveld J, Cornelissen AW, Heck AJ, de Vries E. Comprehensive analysis of the secreted proteins of the parasite Haemonchus contortus reveals extensive sequence variation and differential immune recognition. The Journal of biological chemistry. 2003;278(19):16941–51. Epub 2003/02/11. 10.1074/jbc.M212453200 . 12576473

[17] Anbu KA, Joshi P. Identification of a 55 kDa Haemonchus contortus excretory/secretory glycoprotein as a neutrophil inhibitory factor. Parasite Immunol. 2008;30(1):23–30. 10.1111/j.1365-3024.2007.00995.x 18086013

[18] Reinhardt S, Scott I, Simpson HV. Neutrophil and eosinophil chemotactic factors in the excretory/secretory products of sheep abomasal nematode parasites: NCF and ECF in abomasal nematodes. Parasitology research. 2011;109(3):627–35. Epub 2011/03/23. 10.1007/s00436-011-2305-8 . 21424403

[19] Wang W, Wang S, Zhang H, Yuan C, Yan R, Song X, et al Galectin Hco-gal-m from Haemonchus contortus modulates goat monocytes and T cell function in different patterns. Parasites & vectors. 2014;7:342 Epub 2014/07/25. 10.1186/1756-3305-7-342 ; PubMed Central PMCID: PMCPmc4117971. 25056558 PMC4117971

[20] Merkelbach P, Scott I, Khalaf S, Simpson HV. Excretory/secretory products of Haemonchus contortus inhibit aminopyrine accumulation by rabbit gastric glands in vitro. Veterinary parasitology. 2002;104(3):217–28. Epub 2002/01/29. . 11812620 10.1016/s0304-4017(01)00635-5

[21] Przemeck S, Huber A, Brown S, Pedley KC, Simpson HV. Excretory/secretory products of sheep abomasal nematode parasites cause vacuolation and increased neutral red uptake by HeLa cells. Parasitology research. 2005;95(3):213–7. Epub 2005/02/22. 10.1007/s00436-004-1280-8 . 15723269

[22] Huber A, Prosl H, Joachim A, Simpson HV, Pedley KC. Effects of excretory/secretory products of Haemonchus contortus on cell vacuolation. Parasitology research. 2005;96(5):290–5. Epub 2005/05/27. 10.1007/s00436-005-1321-y . 15918071

[23] Yan F, Xu L, Liu L, Yan R, Song X, Li X. Immunoproteomic analysis of whole proteins from male and female adult Haemonchus contortus. Vet J. 2010;185(2):174–9. Epub 2009/06/30. 10.1016/j.tvjl.2009.05.021 . 19560953

[24] Yatsuda AP, Eysker M, Vieira-Bressan MC, De Vries E. A family of activation associated secreted protein (ASP) homologues of Cooperia punctata. Res Vet Sci. 2002;73(3):297–306. Epub 2002/11/22. . 12443689 10.1016/s0034-5288(02)00125-x

[25] Saverwyns H, Visser A, Nisbet AJ, Peelaers I, Gevaert K, Vercruysse J, et al Identification and characterization of a novel specific secreted protein family for selected members of the subfamily Ostertagiinae (Nematoda). Parasitology. 2008;135(Pt 1):63–70. Epub 2007/10/03. 10.1017/s0031182007003666 . 17908364

[26] Craig H, Wastling JM, Knox DP. A preliminary proteomic survey of the in vitro excretory/secretory products of fourth-stage larval and adult Teladorsagia circumcincta. Parasitology. 2006;132(Pt 4):535–43. Epub 2006/01/04. 10.1017/s0031182005009510 . 16388693

[27] Wang W, Yuan C, Wang S, Song X, Xu L, Yan R, et al Transcriptional and proteomic analysis reveal recombinant galectins of Haemonchus contortus down-regulated functions of goat PBMC and modulation of several signaling cascades in vitro. J Proteomics. 2014;98(0):123–37. 10.1016/j.jprot.2013.12.017. 24401599

[28] Gitt MA, Wiser MF, Leffler H, Herrmann J, Xia YR, Massa SM, et al Sequence and mapping of galectin-5, a beta-galactoside-binding lectin, found in rat erythrocytes. The Journal of biological chemistry. 1995;270(10):5032–8. Epub 1995/03/10. . 7890611 10.1074/jbc.270.10.5032

[29] Kasai K, Hirabayashi J. Galectins: a family of animal lectins that decipher glycocodes. Journal of biochemistry. 1996;119(1):1–8. Epub 1996/01/01. . 8907168 10.1093/oxfordjournals.jbchem.a021192

[30] Gadahi JA, Yongqian B, Ehsan M, Zhang ZC, Wang S, Yan RF, et al Haemonchus contortus excretory and secretory proteins (HcESPs) suppress functions of goat PBMCs in vitro. Oncotarget. 2016 Epub 2016/05/28. 10.18632/oncotarget.9589 . 27229536 PMC5094953

[31] Bradford MM. A rapid and sensitive method for the quantitation of microgram quantities of protein utilizing the principle of protein-dye binding. Anal Biochem. 1976;72:248–54. Epub 1976/05/07. . 942051 10.1016/0003-2697(76)90527-3

[32] Han K, Xu L, Yan R, Song X, Li X. Molecular cloning, expression and characterization of enolase from adult Haemonchus contortus. Research in veterinary science. 2012;92(2):259–65. Epub 2011/03/30. 10.1016/j.rvsc.2011.03.008 . 21444098

[33] Wang Y, Yang W, Cama V, Wang L, Cabrera L, Ortega Y, et al Population genetics of Cryptosporidium meleagridis in humans and birds: evidence for cross-species transmission. Int J Parasitol. 2014;44(8):515–21. Epub 2014/04/15. 10.1016/j.ijpara.2014.03.003 . 24727090

[34] Laing R, Kikuchi T, Martinelli A, Tsai IJ, Beech RN, Redman E, et al The genome and transcriptome of Haemonchus contortus, a key model parasite for drug and vaccine discovery. Genome Biology. 2013;14(8):R88–R. 10.1186/gb-2013-14-8-r88 PMC4054779. 23985316 PMC4054779

[35] Ravooru N, Ganji S, Sathyanarayanan N, Nagendra HG. Insilico analysis of hypothetical proteins unveils putative metabolic pathways and essential genes in Leishmania donovani. Front Genet. 2014;5:291 Epub 2014/09/11. 10.3389/fgene.2014.00291 25206363 PMC4144268

[36] Doerks T, van Noort V, Minguez P, Bork P. Annotation of the M. tuberculosis hypothetical orfeome: adding functional information to more than half of the uncharacterized proteins. PLoS One. 2012;7(4):e34302 Epub 2012/04/10. 10.1371/journal.pone.0034302 22485162 PMC3317503

[37] Szklarczyk D, Franceschini A, Kuhn M, Simonovic M, Roth A, Minguez P, et al The STRING database in 2011: functional interaction networks of proteins, globally integrated and scored. Nucleic Acids Res. 2011;39(Database issue):D561–8. Epub 2010/11/04. 10.1093/nar/gkq973 ; PubMed Central PMCID: PMCPmc3013807. 21045058 PMC3013807

[38] Yan R, Wang J, Xu L, Song X, Li X. DNA vaccine encoding Haemonchus contortus actin induces partial protection in goats. Acta Parasitol. 2014;59(4):698–709. Epub 2014/09/23. 10.2478/s11686-014-0298-z . 25236283

[39] Han K, Xu L, Yan R, Song X, Li X. Cloning, expression and characterization of NAD+-dependent glyceraldehyde-3-phosphate dehydrogenase of adult Haemonchus contortus. J Helminthol. 2011;85(4):421–9. Epub 2011/01/06. 10.1017/s0022149x10000763 . 21205411

[40] Yuan C, Zhang H, Wang W, Li Y, Yan R, Xu L, et al Transmembrane protein 63A is a partner protein of Haemonchus contortus galectin in the regulation of goat peripheral blood mononuclear cells. Parasit Vectors. 2015;8(1):211 Epub 2015/04/17. 10.1186/s13071-015-0816-3 ; PubMed Central PMCID: PMCPmc4404006. 25879191 PMC4404006

[41] Jacobs JR, Sommers KN, Zajac AM, Notter DR, Bowdridge SA. Early IL-4 gene expression in abomasum is associated with resistance to Haemonchus contortus in hair and wool sheep breeds. Parasite immunology. 2016;38(6):333–9. Epub 2016/04/10. 10.1111/pim.12321 . 27059919

[42] Estrada-Reyes ZM, López-Reyes AG, Lagunas-Martínez A, Ramírez- Vargas G, Olazarán-Jenkins S, Hernández-Romano J, et al Relative expression analysis of IL-5 and IL-6 genes in tropical sheep breed Pelibuey infected with Haemonchus contortus. Parasite immunology. 2015;37(9):446–52. 10.1111/pim.12211 26094646

[43] Knox D. Proteases in blood-feeding nematodes and their potential as vaccine candidates. Adv Exp Med Biol. 2011;712:155–76. Epub 2011/06/11. 10.1007/978-1-4419-8414-2_10 . 21660664

[44] Hartman D, Donald DR, Nikolaou S, Savin KW, Hasse D, Presidente PJ, et al Analysis of developmentally regulated genes of the parasite Haemonchus contortus. Int J Parasitol. 2001;31(11):1236–45. Epub 2001/08/22. . 11513893 10.1016/s0020-7519(01)00248-x

[45] Hu Y, Zhang E, Huang L, Li W, Liang P, Wang X, et al Expression profiles of glyceraldehyde-3-phosphate dehydrogenase from Clonorchis sinensis: a glycolytic enzyme with plasminogen binding capacity. Parasitol Res. 2014;113(12):4543–53. Epub 2014/10/11. 10.1007/s00436-014-4144-x . 25300416

[46] Hara MR, Agrawal N, Kim SF, Cascio MB, Fujimuro M, Ozeki Y, et al S-nitrosylated GAPDH initiates apoptotic cell death by nuclear translocation following Siah1 binding. Nat Cell Biol. 2005;7(7):665–74. Epub 2005/06/14. 10.1038/ncb1268 . 15951807

[47] El Ridi R, Tallima H. Vaccine-induced protection against murine schistosomiasis mansoni with larval excretory-secretory antigens and papain or type-2 cytokines. J Parasitol. 2013;99(2):194–202. Epub 2012/09/19. 10.1645/ge-3186.1 . 22985345

[48] Nikolaou S, Hartman D, Nisbet AJ, Presidente PJ, Gasser RB. Genomic organization and expression analysis for hcstk, a serine/threonine protein kinase gene of Haemonchus contortus, and comparison with Caenorhabditis elegans par-1. Gene. 2004;343(2):313–22. Epub 2004/12/14. 10.1016/j.gene.2004.09.017 . 15588586

[49] Ali AS, Ali S, El-Rayes BF, Philip PA, Sarkar FH. Exploitation of protein kinase C: a useful target for cancer therapy. Cancer Treat Rev. 2009;35(1):1–8. Epub 2008/09/10. 10.1016/j.ctrv.2008.07.006 . 18778896

[50] Breugelmans B, Jex AR, Korhonen PK, Mangiola S, Young ND, Sternberg PW, et al Bioinformatic exploration of RIO protein kinases of parasitic and free-living nematodes. Int J Parasitol. 2014;44(11):827–36. Epub 2014/07/20. 10.1016/j.ijpara.2014.06.005 . 25038443

[51] Yuan W, Lok JB, Stoltzfus JD, Gasser RB, Fang F, Lei WQ, et al Toward understanding the functional role of Ss-RIOK-1, a RIO protein kinase-encoding gene of Strongyloides stercoralis. PLoS Negl Trop Dis. 2014;8(8):e3062 Epub 2014/08/08. 10.1371/journal.pntd.0003062 25101874 PMC4125297

[52] Geering B. Death-associated protein kinase 2: Regulator of apoptosis, autophagy and inflammation. Int J Biochem Cell Biol. 2015;65:151–4. Epub 2015/06/10. 10.1016/j.biocel.2015.06.001 . 26055515

[53] Siles-Lucas M. The 14-3-3 protein: a key molecule in parasites as in other organisms. Trends Parasitol. 2003;19(12):575–81. 10.1016/j.pt.2003.10.003 14642768

[54] Wang W, Shakes DC. Molecular evolution of the 14-3-3 protein family. J Mol Evol. 1996;43(4):384–98. Epub 1996/10/01. 10.1007/BF02339012 . 8798343

[55] Jeanclos EM, Lin L, Treuil MW, Rao J, DeCoster MA, Anand R. The chaperone protein 14-3-3eta interacts with the nicotinic acetylcholine receptor alpha 4 subunit. Evidence for a dynamic role in subunit stabilization. J Biol Chem. 2001;276(30):28281–90. Epub 2001/05/16. 10.1074/jbc.M011549200 . 11352901

[56] Koyama T, Ohsawa T, Shimada S, Omata Y, Xuan X, Inoue N, et al A 14-3-3 protein homologue is expressed in feline enteroepithelial-stages of Toxoplasma gondii. Vet Parasitol. 2001;96(1):65–74. 10.1016/S0304-4017(00)00424-6. 11182236

[57] McGonigle S, Beall MJ, Feeney EL, Pearce EJ. Conserved role for 14-3-3epsilon downstream of type I TGFbeta receptors. FEBS Lett. 2001;490(1–2):65–9.doi: citeulike-article-id:3736418. 11172812 10.1016/s0014-5793(01)02133-0

[58] Heusden GPH, Griffiths DJF, Ford JC, Chin-A-Woeng TFC, Schrader PAT, Carr AM, et al The 14-3-3 proteins encoded by the BMH1 and BMH2 genes are essential in the yeast Saccharomyces cerevisiae and can be replaced by a plant homologue. Eur J Biochem. 1995;229(1):45–53. 10.1111/j.1432-1033.1995.0045l.x 7744048

[59] Hoekstra R, Visser A, Otsen M, Tibben J, Lenstra JA, Roos MH. EST sequencing of the parasitic nematode Haemonchus contortus suggests a shift in gene expression during transition to the parasitic stages. Mol Biochem Parasitol. 2000;110(1):53–68. 10.1016/S0166-6851(00)00255-3. 10989145

[60] Aghazadeh Y, Ye X, Blonder J, Papadopoulos V. Protein modifications regulate the role of 14-3-3gamma adaptor protein in cAMP-induced steroidogenesis in MA-10 Leydig cells. J Biol Chem. 2014;289(38):26542–53. Epub 2014/08/03. 10.1074/jbc.M114.569079 25086053 PMC4176220

[61] Jeon YH, Park YH, Kwon JH, Lee JH, Kim IY. Inhibition of 14-3-3 binding to Rictor of mTORC2 for Akt phosphorylation at Ser473 is regulated by selenoprotein W. Biochim Biophys Acta. 2013;1833(10):2135–42. Epub 2013/05/18. 10.1016/j.bbamcr.2013.05.005 . 23680186

[62] Kiss JE, Gao X, Krepp JM, Hawdon JM. Interaction of hookworm 14-3-3 with the forkhead transcription factor DAF-16 requires intact Akt phosphorylation sites. Parasit Vectors. 2009;2:21–. 10.1186/1756-3305-2-21 PMC2683825. 19393088 PMC2683825

[63] Sotillo J, Sanchez-Flores A, Cantacessi C, Harcus Y, Pickering D, Bouchery T, et al Secreted proteomes of different developmental stages of the gastrointestinal nematode Nippostrongylus brasiliensis. Mol Cell Proteomics. 2014;13(10):2736–51. Epub 2014/07/06. 10.1074/mcp.M114.038950 24994561 PMC4188999

[64] Morassutti AL, Levert K, Pinto PM, da Silva AJ, Wilkins P, Graeff-Teixeira C. Characterization of Angiostrongylus cantonensis excretory-secretory proteins as potential diagnostic targets. Exp Parasitol. 2012;130(1):26–31. Epub 2011/10/25. 10.1016/j.exppara.2011.10.003 . 22019415

[65] De Loof A, Vanden J, Janssen I. Hormones and the cytoskeleton of animals and plants. Int Rev Cytol. 1996;166:1–58. Epub 1996/01/01. . 8881772 10.1016/s0074-7696(08)62505-x

[66] Kovaleva ES, Subbotin SA, Masler EP, Chitwood DJ. Molecular characterization of the actin gene from cyst nematodes in comparison with those from other nematodes. Comp Parasitol. 2005;72(1):39–49. 10.1654/4138

[67] Fang L, Sun L, Yang J, Gu Y, Zhan B, Huang J, et al Heat shock protein 70 from Trichinella spiralis induces protective immunity in BALB/c mice by activating dendritic cells. Vaccine. 2014;32(35):4412–9. Epub 2014/06/26. 10.1016/j.vaccine.2014.06.055 . 24962751

[68] Hartmann W, Singh N, Rathaur S, Brenz Y, Liebau E, Fleischer B, et al Immunization with Brugia malayi Hsp70 protects mice against Litomosoides sigmodontis challenge infection. Parasite Immunol. 2014;36(4):141–9. Epub 2013/12/24. 10.1111/pim.12093 . 24359133

[69] Srivastava S, Srikanth E, Liebau E, Rathaur S. Identification of Setaria cervi heat shock protein 70 by mass spectrometry and its evaluation as diagnostic marker for lymphatic filariasis. Vaccine. 2010;28(5):1429–36. Epub 2009/07/04. 10.1016/j.vaccine.2009.06.044 . 19573640

[70] Zhang H, Zhou Q, Yang Y, Chen X, Yan B, Du A. Characterization of heat shock protein 70 gene from Haemonchus contortus and its expression and promoter analysis in Caenorhabditis elegans. Parasitology. 2013;140(6):683–94. Epub 2013/01/31. 10.1017/S0031182012002168 . 23360558

[71] van Leeuwen MA. Heat-shock and stress response of the parasitic nematode Haemonchus contortus. Parasitol Res. 1995;81(8):706–9. Epub 1995/01/01. . 8570590 10.1007/BF00931852

[72] Laing R, Kikuchi T, Martinelli A, Tsai IJ, Beech RN, Redman E, et al The genome and transcriptome of Haemonchus contortus, a key model parasite for drug and vaccine discovery. Genome Biol. 2013;14(8):R88 Epub 2013/08/30. 10.1186/gb-2013-14-8-r88 ; PubMed Central PMCID: PMCPmc4054779. 23985316 PMC4054779

[73] Bennuru S, Semnani R, Meng Z, Ribeiro JM, Veenstra TD, Nutman TB. Brugia malayi excreted/secreted proteins at the host/parasite interface: stage- and gender-specific proteomic profiling. PLoS Negl Trop Dis. 2009;3(4):e410 Epub 2009/04/09. 10.1371/journal.pntd.0000410 ; PubMed Central PMCID: PMCPmc2659452. 19352421 PMC2659452

[74] Hewitson JP, Harcus Y, Murray J, van Agtmaal M, Filbey KJ, Grainger JR, et al Proteomic analysis of secretory products from the model gastrointestinal nematode Heligmosomoides polygyrus reveals dominance of venom allergen-like (VAL) proteins. J Proteomics. 2011;74(9):1573–94. Epub 2011/07/05. 10.1016/j.jprot.2011.06.002 . 21722761 PMC4794625

[75] Yoshino TP, Brown M, Wu XJ, Jackson CJ, Ocadiz-Ruiz R, Chalmers IW, et al Excreted/secreted Schistosoma mansoni venom allergen-like 9 (SmVAL9) modulates host extracellular matrix remodelling gene expression. Int J Parasitol. 2014;44(8):551–63. Epub 2014/05/27. 10.1016/j.ijpara.2014.04.002 24859313 PMC4079936

[76] Murray J, Gregory WF, Gomez-Escobar N, Atmadja AK, Maizels RM. Expression and immune recognition of Brugia malayi VAL-1, a homologue of vespid venom allergens and Ancylostoma secreted proteins. Mol Biochem Parasitol. 2001;118(1):89–96. Epub 2001/11/13. . 11704277 10.1016/s0166-6851(01)00374-7

[77] Rehman A, Jasmer DP. A tissue specific approach for analysis of membrane and secreted protein antigens from Haemonchus contortus gut and its application to diverse nematode species. Mol Biochem Parasitol. 1998;97(1–2):55–68. Epub 1999/01/08. . 9879887 10.1016/s0166-6851(98)00132-7

[78] Vinkenoog R, Speranca MA, van Breemen O, Ramesar J, Williamson DH, Ross-MacDonald PB, et al Malaria parasites contain two identical copies of an elongation factor 1 alpha gene. Mol Biochem Parasitol. 1998;94(1):1–12. . 9719506 10.1016/s0166-6851(98)00035-8

[79] Kaur KJ, Ruben L. Protein translation elongation factor-1 alpha from Trypanosoma brucei binds calmodulin. J Biol Chem. 1994;269(37):23045–50. Epub 1994/09/16. . 8083206

[80] Carlton JM, Hirt RP, Silva JC, Delcher AL, Schatz M, Zhao Q, et al Draft genome sequence of the sexually transmitted pathogen Trichomonas vaginalis. Science. 2007;315(5809):207–12. Epub 2007/01/16. 10.1126/science.1132894 ; PubMed Central PMCID: PMCPmc2080659. 17218520 PMC2080659

[81] Xu P, Widmer G, Wang Y, Ozaki LS, Alves JM, Serrano MG, et al The genome of Cryptosporidium hominis. Nature. 2004;431(7012):1107–12. Epub 2004/10/29. 10.1038/nature02977 . 15510150

[82] Kim T, Cho P, Na J, Hong S-J. Molecular cloning and phylogenetic analysis of Clonorchis sinensis elongation factor-1α. Parasitol Res. 2007;101(6):1557–62. 10.1007/s00436-007-0676-7 17674047

[83] Zamanian M, Fraser LM, Agbedanu PN, Harischandra H, Moorhead AR, Day TA, et al Release of Small RNA-containing Exosome-like Vesicles from the Human Filarial Parasite Brugia malayi. PLoS neglected tropical diseases. 2015;9(9):e0004069 10.1371/journal.pntd.0004069 PMC4581865. 26401956 PMC4581865

[84] Riis B, Rattan SI, Clark BF, Merrick WC. Eukaryotic protein elongation factors. Trends Biochem Sci. 1990;15(11):420–4. . 2278101 10.1016/0968-0004(90)90279-k

[85] Ransom-Hodgkins WD. The application of expression analysis in elucidating the eukaryotic elongation factor one alpha gene family in Arabidopsis thaliana. Mol Genet Genomics. 2009;281(4):391–405. 10.1007/s00438-008-0418-2 . 19132394

[86] Matsubayashi M, Teramoto-Kimata I, Uni S, Lillehoj HS, Matsuda H, Furuya M, et al Elongation factor-1alpha is a novel protein associated with host cell invasion and a potential protective antigen of Cryptosporidium parvum. J Biol Chem. 2013;288(47):34111–20. Epub 2013/10/03. 10.1074/jbc.M113.515544 ; PubMed Central PMCID: PMCPmc3837153. 24085304 PMC3837153

[87] Toueille M, Saint-Jean B, Castroviejo M, Benedetto JP. The elongation factor 1A: a novel regulator in the DNA replication/repair protein network in wheat cells? Plant Physiol Biochem. 2007;45(2):113–8. 10.1016/j.plaphy.2007.01.006 . 17344053

[88] Blanch A, Robinson F, Watson IR, Cheng LS, Irwin MS. Eukaryotic translation elongation factor 1-alpha 1 inhibits p53 and p73 dependent apoptosis and chemotherapy sensitivity. PLoS One. 2013;8(6):e66436 10.1371/journal.pone.0066436 23799104 PMC3682968

[89] Geldhof P, Whitton C, Gregory WF, Blaxter M, Knox DP. Characterisation of the two most abundant genes in the Haemonchus contortus expressed sequence tag dataset. Int J Parasitol. 2005;35(5):513–22. Epub 2005/04/14. 10.1016/j.ijpara.2005.02.009 . 15826643

[90] Rhoads RE, Dinkova TD, Korneeva NL. Mechanism and regulation of translation in C. elegans. WormBook. 2006:1–18. Epub 2007/12/01. 10.1895/wormbook.1.63.1 . 18050488 PMC4781424

[91] Cantacessi C, Campbell BE, Young ND, Jex AR, Hall RS, Presidente PJ, et al Differences in transcription between free-living and CO2-activated third-stage larvae of Haemonchus contortus. BMC Genomics. 2010;11:266 Epub 2010/04/28. 10.1186/1471-2164-11-266 ; PubMed Central PMCID: PMCPmc2880303. 20420710 PMC2880303

[92] Reddien PW, Horvitz HR. CED-2/CrkII and CED-10/Rac control phagocytosis and cell migration in Caenorhabditis elegans. Nat Cell Biol. 2000;2(3):131–6. Epub 2000/03/09. 10.1038/35004000 . 10707082

[93] Ma GX, Zhou RQ, Hu SJ, Huang HC, Zhu T, Xia QY. Molecular characterization and functional analysis of serine/threonine protein phosphatase of Toxocara canis. Exp Parasitol. 2014;141:55–61. Epub 2014/03/25. 10.1016/j.exppara.2014.03.019 . 24657583

[94] Hu M, Abs El-Osta YG, Campbell BE, Boag PR, Nisbet AJ, Beveridge I, et al Trichostrongylus vitrinus (Nematoda: Strongylida): Molecular characterization and transcriptional analysis of Tv-stp-1, a serine/threonine phosphatase gene. Exp Parasitol. 2007;117(1):22–34. 10.1016/j.exppara.2007.03.008. 17490653

[95] Kutuzov MA, Andreeva AV. Protein Ser/Thr phosphatases of parasitic protozoa. Mol Biochem Parasitol. 2008;161(2):81–90. 10.1016/j.molbiopara.2008.06.008. 10.1016/j.molbiopara.2008.06.008 18619495

[96] Campbell BE, Hofmann A, McCluskey A, Gasser RB. Serine/threonine phosphatases in socioeconomically important parasitic nematodes—prospects as novel drug targets? Biotechnol Adv. 2011;29(1):28–39. Epub 2010/08/25. 10.1016/j.biotechadv.2010.08.008 . 20732402

[97] Klumpp S, Krieglstein J. Serine/threonine protein phosphatases in apoptosis. Curr Opin Pharmacol. 2002;2(4):458–62. Epub 2002/07/20. . 12127881 10.1016/s1471-4892(02)00176-5

[98] Berndt N. Roles and regulation of serine/threonine-specific protein phosphatases in the cell cycle. Prog Cell Cycle Res. 2003;5:497–510. Epub 2003/11/05. . 14593745

[99] Sarmiento M, Zhao Y, Gordon SJ, Zhang ZY. Molecular basis for substrate specificity of protein-tyrosine phosphatase 1B. J Biol Chem. 1998;273(41):26368–74. Epub 1998/10/03. . 9756867 10.1074/jbc.273.41.26368

[100] Verpelli C, Schmeisser MJ, Sala C, Boeckers TM. Scaffold proteins at the postsynaptic density In: Kreutz MR, Sala C, editors. Synaptic plasticity: dynamics, development and disease. New York: Springer-Verlag; 2012 p. 29–58. 10.1007/978-3-7091-0932-8_2 22351050

[101] Ferrell JE. What do scaffold proteins really do? Sci STKE. 2000;2000:pe1. 10.1126/stke.522000pe1 11752612

[102] Good MC, Zalatan JG, Lim WA. Scaffold proteins: hubs for controlling the flow of cellular information. Science. 2011;332(6030):680–6. Epub 2011/05/10. 10.1126/science.1198701 21551057 PMC3117218

[103] Bray D, Lay S. Computer-based analysis of the binding steps in protein complex formation. Proc Natl Acad Sci U S A. 1997;94(25):13493–8. Epub 1998/02/12. ; PubMed Central PMCID: PMCPmc28333. 9391053 10.1073/pnas.94.25.13493 PMC28333

[104] Levchenko A, Bruck J, Sternberg PW. Scaffold proteins may biphasically affect the levels of mitogen-activated protein kinase signaling and reduce its threshold properties. Proc Natl Acad Sci U S A. 2000;97(11):5818–23. Epub 2000/05/24. ; PubMed Central PMCID: PMCPmc18517. 10823939 10.1073/pnas.97.11.5818 PMC18517

[105] Nie Z, Hirsch DS, Randazzo PA. Arf and its many interactors. Curr Opin Cell Biol. 2003;15(4):396–404. Epub 2003/08/02. . 12892779 10.1016/s0955-0674(03)00071-1

[106] Ackema KB, Hench J, Bockler S, Wang SC, Sauder U, Mergentaler H, et al The small GTPase Arf1 modulates mitochondrial morphology and function. EMBO J. 2014;33(22):2659–75. Epub 2014/09/06. 10.15252/embj.201489039 ; PubMed Central PMCID: PMCPmc4282574. 25190516 PMC4282574

[107] Singhvi A, Teuliere J, Talavera K, Cordes S, Ou G, Vale RD, et al The Arf GAP CNT-2 regulates the apoptotic fate in C. elegans asymmetric neuroblast divisions. Curr Biol. 2011;21(11):948–54. Epub 2011/05/21. 10.1016/j.cub.2011.04.025 ; PubMed Central PMCID: PMCPmc3109113. 21596567 PMC3109113

[108] Issouf M, Guegnard F, Koch C, Le Vern Y, Blanchard-Letort A, Che H, et al Haemonchus contortus P-glycoproteins interact with host eosinophil granules: a novel insight into the role of ABC transporters in host-parasite interaction. PLoS ONE. 2014;9(2):e87802 Epub 2014/02/06. 10.1371/journal.pone.0087802 ; PubMed Central PMCID: PMCPmc3912070. 24498376 PMC3912070

[109] Williamson SM, Wolstenholme AJ. P-glycoproteins of Haemonchus contortus: development of real-time PCR assays for gene expression studies. J Helminthol. 2012;86(2):202–8. Epub 2011/07/07. 10.1017/s0022149x11000216 . 21729384

[110] Valadi H, Ekstrom K, Bossios A, Sjostrand M, Lee JJ, Lotvall JO. Exosome-mediated transfer of mRNAs and microRNAs is a novel mechanism of genetic exchange between cells. Nature cell biology. 2007;9(6):654–9. Epub 2007/05/09. 10.1038/ncb1596 . 17486113

[111] Buck AH, Coakley G, Simbari F, McSorley HJ, Quintana JF, Le Bihan T, et al Exosomes secreted by nematode parasites transfer small RNAs to mammalian cells and modulate innate immunity. Nature communications. 2014;5:5488 Epub 2014/11/26. 10.1038/ncomms6488 ; PubMed Central PMCID: PMCPmc4263141. 25421927 PMC4263141

[112] Barteneva NS, Maltsev N, Vorobjev IA. Microvesicles and intercellular communication in the context of parasitism. Frontiers in cellular and infection microbiology. 2013;3:49 Epub 2013/09/14. 10.3389/fcimb.2013.00049 ; PubMed Central PMCID: PMCPmc3764926. 24032108 PMC3764926

[113] Silverman JM, Clos J, de'Oliveira CC, Shirvani O, Fang Y, Wang C, et al An exosome-based secretion pathway is responsible for protein export from Leishmania and communication with macrophages. Journal of cell science. 2010;123(Pt 6):842–52. Epub 2010/02/18. 10.1242/jcs.056465 . 20159964

[114] Nowacki FC, Swain MT, Klychnikov OI, Niazi U, Ivens A, Quintana JF, et al Protein and small non-coding RNA-enriched extracellular vesicles are released by the pathogenic blood fluke Schistosoma mansoni. Journal of extracellular vesicles. 2015;4:28665 Epub 2015/10/08. 10.3402/jev.v4.28665 ; PubMed Central PMCID: PMCPmc4595467. 26443722 PMC4595467

[115] Sotillo J, Pearson M, Becker L, Mulvenna J, Loukas A. A quantitative proteomic analysis of the tegumental proteins from Schistosoma mansoni schistosomula reveals novel potential therapeutic targets. International journal for parasitology. 2015;45(8):505–16. Epub 2015/04/26. 10.1016/j.ijpara.2015.03.004 . 25910674

[116] Buck AH, Coakley G, Simbari F, McSorley HJ, Quintana JF, Le Bihan T, et al Exosomes secreted by nematode parasites transfer small RNAs to mammalian cells and modulate innate immunity. Nature communications. 2014;5:5488 10.1038/ncomms6488 PMC4263141. 25421927 PMC4263141

[117] Marcilla A, Trelis M, Cortes A, Sotillo J, Cantalapiedra F, Minguez MT, et al Extracellular vesicles from parasitic helminths contain specific excretory/secretory proteins and are internalized in intestinal host cells. PLoS One. 2012;7(9):e45974 Epub 2012/10/03. 10.1371/journal.pone.0045974 ; PubMed Central PMCID: PMCPmc3454434. 23029346 PMC3454434

[118] Moreno Y, Gros PP, Tam M, Segura M, Valanparambil R, Geary TG, et al Proteomic analysis of excretory-secretory products of Heligmosomoides polygyrus assessed with next-generation sequencing transcriptomic information. PLoS Negl Trop Dis. 2011;5(10):e1370 Epub 2011/11/01. 10.1371/journal.pntd.0001370 ; PubMed Central PMCID: PMCPmc3201918. 22039562 PMC3201918

[119] Morphew RM, Wright HA, LaCourse EJ, Woods DJ, Brophy PM. Comparative proteomics of excretory-secretory proteins released by the liver fluke Fasciola hepatica in sheep host bile and during in vitro culture ex host. Mol Cell Proteomics. 2007;6(6):963–72. Epub 2007/02/20. 10.1074/mcp.M600375-MCP200 . 17308300

[120] Salavati R, Najafabadi HS. Sequence-based functional annotation: what if most of the genes are unique to a genome? Trends Parasitol. 2010;26(5):225–9. Epub 2010/03/10. 10.1016/j.pt.2010.02.001 . 20211583

[121] Mangiola S, Young ND, Korhonen P, Mondal A, Scheerlinck JP, Sternberg PW, et al Getting the most out of parasitic helminth transcriptomes using HelmDB: implications for biology and biotechnology. Biotechnol Adv. 2013;31(8):1109–19. Epub 2012/12/26. 10.1016/j.biotechadv.2012.12.004 . 23266393

