# Supplementary figures and images for: Proteomic Analysis of the Excretory and Secretory Proteins of Haemonchus contortus (HcESP) Binding to Goat PBMCs In Vivo Revealed Stage-Specific Binding Profiles
Source: PLoS One. 2016 Jul 28;11(7):e0159796. doi: 10.1371/journal.pone.0159796 (PMC4965049; doi:10.1371/journal.pone.0159796)

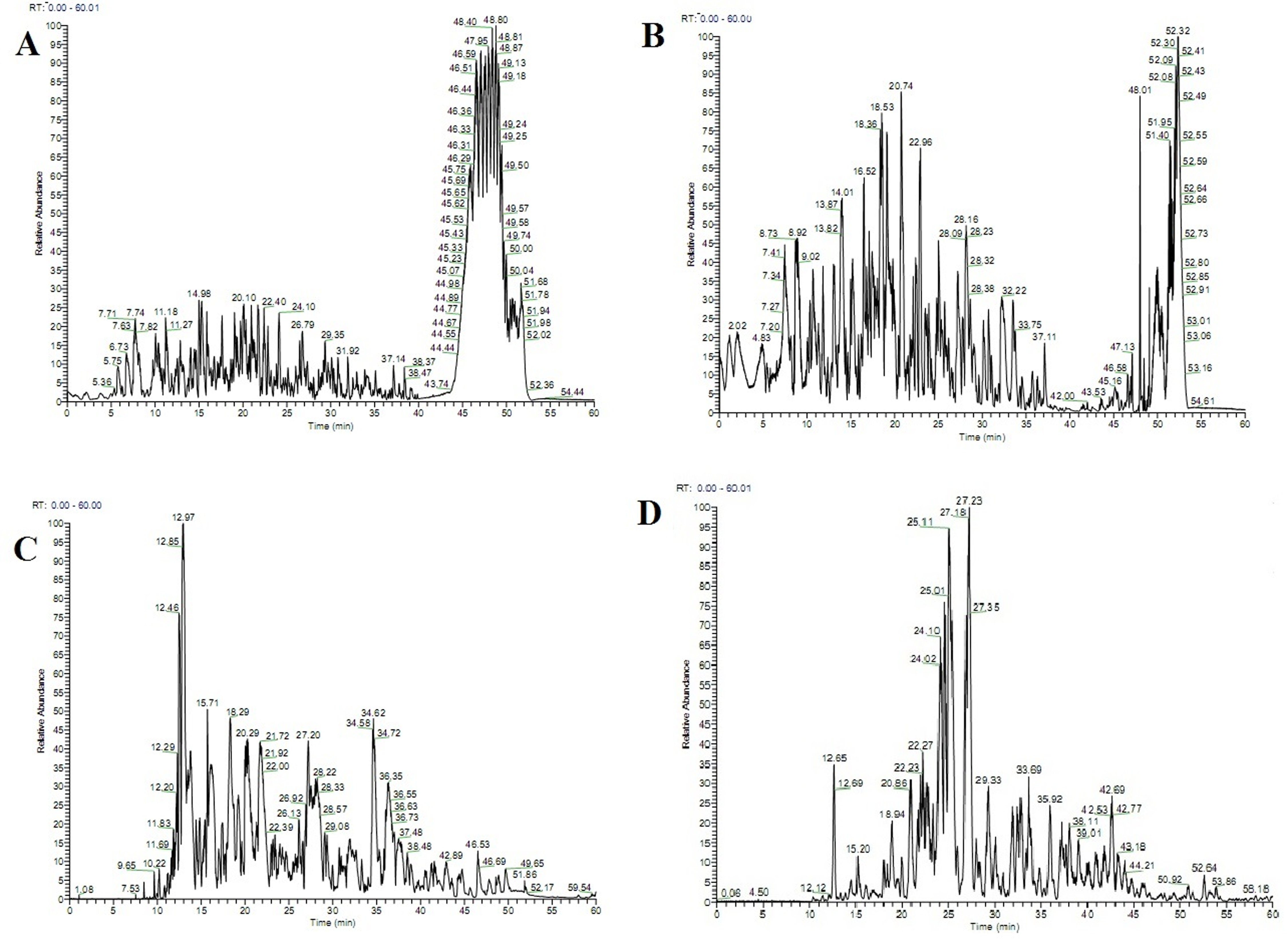

Supplement: S1 Fig — Total ion flow diagram of HcESPs interacted with goat PBMCs at the (A) L4, (B) L5, (C) early adult and (D) late adult stages. (TIF) [file pone.0159796.s001.tif]
