# Supplementary material for: Proteomic Analysis of the Excretory and Secretory Proteins of Haemonchus contortus (HcESP) Binding to Goat PBMCs In Vivo Revealed Stage-Specific Binding Profiles
Source: PLoS One. 2016 Jul 28;11(7):e0159796. doi: 10.1371/journal.pone.0159796 (PMC4965049; doi:10.1371/journal.pone.0159796)
Supplement: S1 Table — (DOCX) [file pone.0159796.s002.docx]

**S1 Table:** List of identified proteins of *HcESP* binding to goat PBMCs shared among different developmental stages *in vivo*

|  | **Protein Description** | **Accession**  **Number** | **Cover %** | **Theoretical**  **Molecular mass (Da)** | **Theoretical**  **iso electrical point** | **Developmental stages** | | | |
| --- | --- | --- | --- | --- | --- | --- | --- | --- | --- |
|  |  |  |  |  |  | **L_4_** | **L_5_** | **Early adult** | **Late adult** |
|  | Actin | U6NLR5 | 45.21 | 41777.28 | 5.30 | + | + | + | + |
|  | Actin | U6PYM7 | 26.93 | 42001.54 | 5.37 | + | + | + | + |
|  | Actin | U6PTS0 | 34.38 | 39162.46 | 5.19 | + | + | + | + |
|  | Tubulin FtsZ | U6P476 | 16.00 | 50055.96 | 5.05 | + | + | + | + |
|  | Myosin and Myosin head and IQ calmodulin-binding region and Myosin tail domain | U6PXP8 | 2.85 | 229436.7 | 5.54 | + | + | + | + |
|  | Beta-tubulin isotype 2 | C5J0J8 | 16.74 | 49784.57 | 4.85 | + | + | + | + |
|  | Heat shock protein 70 | I6MZD7 | 9.29 | 70335.59 | 5.50 | + | + | + | + |
|  | Tubulin FtsZ | U6P3I2 | 10.22 | 50110.07 | 5.00 | + | + | + | + |
|  | Myosin and Myosin | U6PSH3 | 1.67 | 226983.6 | 5.67 | + | + | + | + |
|  | Tubulin FtsZ | U6NVZ7 | 5.37 | 49895.18 | 5.10 | + | + | + | + |
|  | EF hand | W6NI29 | 21.76 | 19788.85 | 4.66 | + | + | + | + |
|  | Spectrin repeat and EF hand | U6NKU0 | 0.86 | 163531.7 | 5.32 | + | + | + | + |
|  | Actin actin | U6PH81 | 3.05 | 44026.62 | 5.46 | + | + | + | + |
|  | Glycoside hydrolase | U6PYZ5 | 1.38 | 75601.11 | 6.01 | + | + | + | + |
|  | Glyceraldehyde-3-phosphate dehydrogenase | D9IL10 | 4.40 | 36446.27 | 8.15 | + | + | + | + |
|  | Zinc finger and FY-rich and SET domain | U6NFY1 | 0.35 | 255064.4 | 6.31 | + | + | + | + |
|  | Zinc finger and FY-rich and SET domain | U6PK91 | 0.38 | 232284.4 | 6.04 | + | + | + | + |
|  | Peptidase S9 | U6NTV7 | 1.22 | 82284.08 | 6.01 | + | + | + | + |
|  | EF hand domain | U6NU05 | 3.18 | 24689.28 | 4.31 | + | + | + | + |
|  | Zinc finger and Nuclear hormone receptor | U6NVP3 | 1.89 | 46854.42 | 7.87 | + | + | + | + |
|  | Zinc finger domain | U6P2R3 | 0.53 | 125337.4 | 8.81 | + | + | + | + |
|  | Zinc finger domain | U6P2Z1 | 1.14 | 76568.19 | 8.82 | + | + | + | + |
|  | Zinc finger domain | U6P3B1 | 0.89 | 101490.2 | 6.25 | + | + | + | + |
|  | RNA-directed DNA polymerase | U6P4S4 | 1.37 | 75129.68 | 9.38 | + | + | + | + |
|  | Zinc finger | U6P6W6 | 1.28 | 71204.68 | 4.99 | + | + | + | + |
|  | Zinc finger | U6P7I1 | 1.39 | 48087.92 | 5.45 | + | + | + | + |
|  | EF hand | U6P8A8 | 3.81 | 49410.33 | 7.63 | + | + | + | + |
|  | Zinc finger and PADR1 and WGR and Poly(ADP-ribose) polymerase domain containing protein | U6PBB0 | 1.66 | 102615 | 7.71 | + | + | + | + |
|  | RNA-directed DNA polymerase | U6PEY2 | 4.23 | 21274.09 | 5.31 | + | + | + | + |
|  | Cyclin | U6PHR7 | 1.42 | 55880.44 | 5.89 | + | + | + | + |
|  | RNA-directed DNA polymerase | U6PJP4 | 2.75 | 33903.31 | 9.83 | + | + | + | + |
|  | Peptidase C46 | U6PKP0 | 0.84 | 120164.7 | 6.41 | + | + | + | + |
|  | Zinc finger | U6PNW2 | 2.20 | 46575.67 | 7.81 | + | + | + | + |
|  | Peptidase S28 | U6PPE8 | 0.65 | 87147.63 | 5.69 | + | + | + | + |
|  | RNA-directed DNA polymerase | U6PPS6 | 0.91 | 74069.71 | 9.81 | + | + | + | + |
|  | Glycoside hydrolase | U6PSA3 | 0.37 | 184198.3 | 6.32 | + | + | + | + |
|  | Peptidase M13 | U6PUB3 | 0.73 | 91273.74 | 5.30 | + | + | + | + |
|  | Zinc finger | U6PVT3 | 1.24 | 63172.47 | 9.24 | + | + | + | + |
|  | Ras domain | U6PY91 | 4.63 | 24337.64 | 6.96 | + | + | + | + |
|  | Actin actin | W6NEU0 | 2.01 | 39481.83 | 5.95 | + | + | + | + |
|  | RNA-directed DNA polymerase | W6NNE9 | 2.65 | 25790.49 | 9.02 | + | + | + | + |
|  | Ras domain | W6NXI7 | 10.20 | 10923.08 | 4.33 | + | + | + | + |
|  | Uncharacterized protein | U6PII4 | 2.25 | 44082.41 | 8.00 | + | + | + | + |
|  | Serine threonine protein kinase | U6P7N4 | 1.26 | 62487.36 | 7.09 | + | + | + | + |
|  | Serine threonine protein kinase | U6PBU1 | 0.37 | 332077.3 | 5.53 | + | + | + | + |
|  | Serine threonine protein kinase | U6PDB2 | 0.39 | 167386.5 | 5.99 | + | + | + | + |
|  | 14-3-3 | U6PP12 | 4.02 | 28261.33 | 4.78 | + | + | + | + |
|  | Beta-tubulin isotype-4 | U5F0E7 | 12.13 | 49972.73 | 4.79 | + | + | + | - |
|  | Elongation factor 1-alpha | U6NYV7 | 4.09 | 50565.6 | 9.08 | + | + | + | - |
|  | ATP synthase subunit alpha | U6PV48 | 3.16 | 57946.28 | 9.08 | + | + | + | - |
|  | ISE/inbred ISE genomic scaffold, scaffold_pathogens_Hcontortus_scaffold_1687 | U6NRB0 | 2.64 | 29185.19 | 4.53 | + | + | + | - |
|  | ISE/inbred ISE genomic scaffold, scaffold_pathogens_Hcontortus_scaffold_1493 | U6NTH3 | 1.15 | 98891.78 | 5.77 | + | + | + | - |
|  | Mitochondrial substrate solute carrier | U6NYP1 | 2.43 | 36219.83 | 9.83 | + | + | + | - |
|  | 40S ribosomal protein S8 | U6P6V7 | 3.37 | 23639 | 10.47 | + | + | + | - |
|  | von Willebrand factor | U6PAI2 | 1.56 | 71323.15 | 6.52 | + | + | + | - |
|  | Calponin actin-binding and Spectrin repeat and Src homology-3 and Pleckstrin homology | U6PE02 | 0.22 | 462861.9 | 6.09 | + | + | + | - |
|  | Glutaredoxin | U6PQG1 | 3.14 | 26313.18 | 9.06 | + | + | + | - |
|  | Ribosomal protein L2 | U6PI85 | 3.08 | 28034.22 | 10.75 | + | + | + |  |
|  | Tumour susceptibility gene 101 and Steadiness box and Ras | U6Q0V4 | 1.75 | 63801.35 | 8.26 | + | + | + | - |
|  | Ras domain containing protein | W6NGM6 | 5.51 | 14496.46 | 8.41 | + | + | + | - |
|  | Ribosomal protein S5 | W6NQF7 | 4.12 | 28527.54 | 10.10 | + | + | + | - |
|  | Histone H2B | U6PWB9 | 13.01 | 13640.58 | 10.43 | + | + | - | + |
|  | Protein-tyrosine phosphatase | U6NUL7 | 1.86 | 43807.38 | 9.30 | + | + | - | + |
|  | Hcontortus_contig_pathogens_Hcontortus_scaffold_5940 | W6NGL8 | 2.91 | 24156.95 | 8.79 | + | + | - | + |
|  | ATPase associated with various cellular activities domain containing protein | U6NHU3 | 0.33 | 204074.9 | 6.33 | + | + | - | + |
|  | SCP extracellular domain containing protein | U6NYV8 | 1.25 | 51521.97 | 5.91 | + | + | - | + |
|  | C. briggsae CBR-UNC-82 | U6NZK6 | 1.45 | 54635.09 | 9.45 | + | + | - | + |
|  | scaffold_pathogens_Hcontortus_scaffold_3144 | U6PBJ7 | 4.42 | 20504.31 | 6.15 | + | + | - | + |
|  | Paired box protein and Transposase domain containing protein | U6PCS3 | 5.96 | 16622.23 | 11.9 | + | + | - | + |
|  | Transposase domain containing protein | U6PIJ6 | 3.21 | 39000.81 | 10.6 | + | + | - | + |
|  | ATPase | U6PK35 | 2.21 | 45519.47 | 5.55 | + | + | - | + |
|  | scaffold_pathogens_Hcontortus_scaffold_859 | U6PX36 | 4.08 | 22822.42 | 7.57 | + | + | - | + |
|  | Uncharacterized protein | W6NR83 | 1.62 | 48313.84 | 5.99 | + | + | - | + |
|  | Cytochrome c oxidase copper chaperone and Sec20 and Ankyrin domain containing protein | U6NZ29 | 1.09 | 61591.55 | 7.30 | + | - | + | + |
|  | DEAD2 domain containing protein | U6PG84 | 0.77 | 102213.6 | 8.35 | + | - | + | + |
|  | Histone H3 | U6PZ56 | 4.41 | 15327.72 | 11.27 | - | + | + | + |
|  | Biotin lipoyl attachment and 2-oxoacid dehydrogenase acyltransferase | U6PB18 | 1.69 | 51160.78 | 9.24 | - | + | + | + |
|  | FAD dependent oxidoreductase and Glycine cleavage T-protein | U6PE59 | 0.90 | 87326.04 | 6.10 | - | + | + | + |
|  | Uncharacterized protein | U6PII4 | 2.25 | 44082.41 | 8.00 | - | + | + | + |
|  | Uncharacterized protein | U6PMT4 | 1.52 | 109013.4 | 6.07 | - | + | + | + |
|  | Histone H2A | U6PU35 | 7.09 | 13440.42 | 10.7 | - | + | + | + |
|  | Tropomyosin domain | U6PH16 | 2.98 | 35033.43 | 4.69 | + | + | - | - |
|  | scaffold_pathogens_Hcontortus_scaffold_1 | U6NEK1 | 2.55 | 25517.1 | 7.09 | + | + | - | - |
|  | Hepatocellular carcinoma-associated antigen 59 | U6NHN1 | 6.38 | 15898.07 | 6.91 | + | + | - | - |
|  | scaffold_pathogens_Hcontortus_scaffold_1223 | U6NP15 | 0.97 | 69094.61 | 8.67 | + | + | - | - |
|  | Immunoglobulin and Immunoglobulin I-set and Fibronectin | U6NSU7 | 0.76 | 147306 | 5.53 | + | + | - | - |
|  | Adenylyl cyclase class-3 4 guanylyl cyclase | U6NXD3 |  |  |  | + | + | - | - |
|  | Endonuclease exonuclease phosphatase | U6P024 |  |  |  | + | + | - | - |
|  | CRE-ACL-6 | U6P0H6 | 1.60 | 42423.34 | 5.63 | + | + | - | - |
|  | Methyltransferase type 12 | U6P1M9 | 2.59 | 35927.32 | 6.47 | + | + | - | - |
|  | Guanine nucleotide exchange factor | U6P5P5 | 2.22 | 60011.61 | 5 | + | + | - | - |
|  | scaffold_pathogens_Hcontortus_scaffold_2698 | U6P5Z8 | 1.35 | 67226.31 | 6.37 | + | + | - | - |
|  | Adenylyl cyclase class-3 4 guanylyl cyclase | U6PJ49 | 0.99 | 90282.92 | 7.54 | + | + | - | - |
|  | CRE-ANC-1 protein (Fragment) | U6PRY3 | 0.64 | 123882.5 | 4.69 | + | + | - | - |
|  | Transcription factor E2F dimerisation partner (TDP) | U6PXX8 | 2.60 | 43725.34 | 5.5 | + | + | - | - |
|  | Scaffold_pathogens_Hcontortus_scaffold_960 | U6Q0Q5 | 3.72 | 21830.81 | 9.43 | + | + | - | - |
|  | Tenascin-like | W6NEW1 | 2.37 | 28841.95 | 8.7 | + | + | - | - |
|  | Cytochrome b5 | W6NG29 | 2.43 | 27705.17 | 8.75 | + | + | - | - |
|  | Ion transport and Potassium channel | W6NHJ5 | 2.49 | 50000.34 | 5.69 | + | + | - | - |
|  | Uncharacterized protein | U6NT62 | 0.71 | 145845.1 | 6.19 | + | + | - | - |
|  | Uncharacterized protein | U6PQ04 | 0.14 | 571660.4 | 5.08 | + | + | - | - |
|  | Tetratricopeptide repeat protein 30A-like | U6PIG7 | 1.01 | 69029.55 | 5.03 | + | - | + | - |
|  | Filament and Basic leucine zipper and Intermediate filament | U6NK71 | 1.22 | 66502.96 | 6.05 | + | - | + | - |
|  | HEAT domain containing protein | U6P692 | 9.29 | 70335.59 | 5.5 | + | - | + | - |
|  | Patched domain containing protein | U6P8Q8 | 4.41 | 15127.73 | 8.46 | + | - | + | - |
|  | LMBR1 conserved region and Mitochondrial inner membrane translocase complex | U6PCU6 | 0.82 | 96677.58 | 8.72 | + | - | + | - |
|  | K Homology | U6PJM2 | 1.83 | 35348.62 | 9.17 | + | - | + | - |
|  | Arp2 3 complex | U6Q073 | 3.00 | 34385.73 | 8.6 | + | - | + | - |
|  | Ion transport | U6NT28 | 0.62 | 130873.8 | 8.84 | + | - | - | + |
|  | RNA recognition motif | U6NUL2 | 0.80 | 99065.57 | 8.75 | + | - | - | + |
|  | Ion transport domain | U6NVT5 | 0.37 | 186108.3 | 7.41 | + | - | - | + |
|  | Scaffold_pathogens_Hcontortus_scaffold_211 | U6P3V2 | 17.39 | 7570.11 | 11.26 | + | - | - | + |
|  | RNA recognition motif | U6P8A9 | 2.46 | 32000.63 | 10.14 | + | - | - | + |
|  | Carboxylesterase | U6PC94 | 1.16 | 58878.51 | 5.8 | + | - | - | + |
|  | Crotonase | U6PPH5 | 2.71 | 28873.95 | 6.6 | + | - | - | + |
|  | RNA recognition motif | U6PRJ8 | 4.07 | 27167.53 | 9.92 | + | - | - | + |
|  | Kinetochore protein Ndc80 | U6PYX6 | 1.15 | 69742.77 | 5.02 | + | - | - | + |
|  | Uncharacterized protein | U6P0H7 | 2.61 | 46514.81 | 9.66 | + | - | - | + |
|  | Nematode cuticle collagen  and Collagen triple helix | W6NQT2 | 3.26 | 34813.68 | 8.93 | + | - | - | + |
|  | Ubiquitin | U6P8E4 | 1.48 | 68297.59 | 7.13 | - | + | + | - |
|  | Major sperm protein | U6NN74 | 2.88 | 39640.52 | 8.29 | - | + | + | - |
|  | 3'-5' exonuclease domain containing | U6NM21 | 1.38 | 64916.05 | 5.98 | - | + | + | - |
|  | Mbt repeat | U6NI50 | 0.86 | 89479.97 | 6.81 | - | + | + | - |
|  | Na+ channel domain containing | U6P0U8 | 5.52 | 18301.42 | 5.08 | - | + | + | - |
|  | NADH:ubiquinone oxidoreductase domain containing protein | U6P5L0 | 6.21 | 17252.52 | 9.2 | - | + | + | - |
|  | Splicing factor PWI | U6P7X9 | 1.57 | 67332.68 | 6.55 | - | + | + | - |
|  | Speract scavenger receptor | U6PQX6 | 0.21 | 323190.3 | 7.12 | - | + | + | - |
|  | Dynein light intermediate chain | U6PY24 | 3.22 | 47310.94 | 4.92 | - | + | + | - |
|  | Scaffold_pathogens_Hcontortus_scaffold_135 | U6NM87 | 0.28 | 288944.2 | 4.8 | - | + | - | + |
|  | CS domain containing | U6NSR1 | 2.48 | 22769.6 | 4.42 | - | + | - | + |
|  | Glycosyl transferase | U6PE24 | 2.40 | 29151.35 | 9.14 | - | + | - | + |
|  | Cadherin and Laminin G | U6PH22 | 0.59 | 208023.1 | 4.85 | - | + | - | + |
|  | SNF2-related and DNA RNA helicase | U6PNS9 | 0.75 | 105900.3 | 6.41 | - | + | - | + |
|  | Protein Y75B8A.8 | U6PP16 | 1.26 | 69812.98 | 7.3 | - | + | - | + |
|  | DNA RNA helicase | U6PYD6 | 1.49 | 67850.98 | 9.49 | - | + | - | + |
|  | Aminoacyl-tRNA synthetase and Valyl Leucyl Isoleucyl-tRNA synthetase | U6PZ22 | 0.65 | 139148.5 | 5.56 | - | + | - | + |
|  | FAD-dependent pyridine nucleotide-disulphide oxidoreductase and Electron transfer flavoprotein-ubiquinone oxidoreductase | W6NPW4 | 1.18 | 66332.18 | 7.6 | - | + | - | + |
|  | Arginyl tRNA synthetase and Arginyl-tRNA synthetase and DALR anticodon binding | U6PXK8 | 1.39 | 80478.43 | 6.54 | - | - | + | + |
|  | Forkhead-associated | W6NTG4 | 1.00 | 104571.2 | 10.23 | - | - | + | + |
|  | Uncharacterized protein | U6NL27 | 2.55 | 31103.15 | 9.29 | - | - | + | + |
|  | Serine/threonine-protein phosphatase | U6P8C9 | 5.21 | 36888.06 | 5.78 | - | - | + | + |
