# Supplementary material for: Proteomic Analysis of the Excretory and Secretory Proteins of Haemonchus contortus (HcESP) Binding to Goat PBMCs In Vivo Revealed Stage-Specific Binding Profiles
Source: PLoS One. 2016 Jul 28;11(7):e0159796. doi: 10.1371/journal.pone.0159796 (PMC4965049; doi:10.1371/journal.pone.0159796)
Supplement: S2 Table — (DOCX) [file pone.0159796.s003.docx]

**S2 Table:** List of developmental stage specific proteins of *HcESP* binding to goat PBMCs identified at different time points *in vivo*

|  | **Protein Description** | **Accession**  **Number** | **Cover**  **%** | **Theoretical**  **Molecular mass (Da)** | **Theoretical**  **iso electrical point** | **Developmental**  **stages** |
| --- | --- | --- | --- | --- | --- | --- |
|  | HAECO Heat shock protein 90 | C7B179 | 3.68 | 81165.17 | 5.03 | L_4_ |
|  | Uncharacterized protein 1 | U6PLW2 | 5.34 | 30499.73 | 9.79 | L_4_ |
|  | Extracellular ligand-binding receptor | U6PRX4 | 1.91 | 47586.22 | 9.21 | L_4_ |
|  | Uncharacterized protein | W6NH38 | 3.19 | 45801.4 | 6.12 | L_4_ |
|  | Protein Y75B8A.24 | U6PA94 | 1.09 | 70432.49 | 7.81 | L_4_ |
|  | Uncharacterized protein | U6PN49 | 0.88 | 90040.34 | 6.02 | L_4_ |
|  | MATH domain containing protein | U6NIA2 | 0.42 | 160001.6 | 5.9 | L_4_ |
|  | Aldehyde dehydrogenase | U6NIW8 | 1.28 | 60118.9 | 6.77 | L_4_ |
|  | Uncharacterized protein | U6NK13 | 4.12 | 19873.08 | 9.87 | L_4_ |
|  | Dbl homology (DH) domain containing protein | U6NKJ3 | 1.18 | 67859.21 | 8.84 | L_4_ |
|  | EAP30 domain containing protein | U6NLN1 | 1.81 | 43267.7 | 6.66 | L_4_ |
|  | scaffold_pathogens_Hcontortus_scaffold_1184 | U6NLP9 | 0.95 | 93062.73 | 5.77 | L_4_ |
|  | PAS fold and Ion transport and Cyclic nucleotide-binding domain containing protein | U6NLY9 | 1.16 | 108072.1 | 8.78 | L_4_ |
|  | Uncharacterized protein | U6NM23 | 3.64 | 38281.84 | 9.59 | L_4_ |
|  | HAD-superfamily hydrolase | U6NM58 | 2.62 | 34408.07 | 5.21 | L_4_ |
|  | Protein ZIM-1, isoform a | U6NMU2 | 1.53 | 64583.85 | 5.16 | L_4_ |
|  | scaffold_pathogens_Hcontortus_scaffold_139 | U6NMX2 | 4.40 | 18433.4 | 9.05 | L_4_ |
|  | Beta-sarcoglycan-like | U6NN55 | 3.74 | 32272.78 | 9.16 | L_4_ |
|  | Low density lipoprotein-receptor | U6NN67 | 7.25 | 15601.75 | 7.48 | L_4_ |
|  | Vinculin alpha-catenin | U6NN69 | 0.90 | 86278.59 | 6.12 | L_4_ |
|  | Rhodanese | U6NN75 | 1.35 | 50622.12 | 8.64 | L_4_ |
|  | Protein T12G3.2, isoform b | U6NNG2 | 3.32 | 23739.83 | 5.73 | L_4_ |
|  | RESA-like protein with DnaJ domain, putative | U6NPE8 | 2.93 | 31093.3 | 5.15 | L_4_ |
|  | Protein FRPR-7 | U6NPL9 | 1.92 | 41286.24 | 6.3 | L_4_ |
|  | Uncharacterized protein | U6NQ56 | 2.79 | 29335.35 | 9.38 | L_4_ |
|  | Condensin complex subunit 1 isoform 2 | U6NQJ1 | 0.40 | 171515.1 | 5.11 | L_4_ |
|  | Cell division cycle protein 16 | U6NRL2 | 1.58 | 73316.15 | 6.31 | L_4_ |
|  | SET domain and mariner transposase fusion protein | U6NRS1 | 4.70 | 16960.29 | 9.73 | L_4_ |
|  | Carbohydrate kinase | U6NRV6 | 1.91 | 45995.94 | 6.76 | L_4_ |
|  | 7TM GPCR | U6NRX3 | 2.75 | 40519.15 | 8.82 | L_4_ |
|  | UPF0516 protein C12orf72 homolog | U6NSZ7 | 4.06 | 22227.95 | 5.59 | L_4_ |
|  | Uncharacterized protein LOC100892795 | U6NT67 | 4.69 | 24591.04 | 10.48 | L_4_ |
|  | Scaffold_pathogens_Hcontortus_scaffold_1870 | U6NV01 | 8.00 | 8671.33 | 8.91 | L_4_ |
|  | Scaffold_pathogens_Hcontortus_scaffold_2012 | U6NVN0 | 0.91 | 72639.87 | 4.91 | L_4_ |
|  | Bromodomain domain containing protein | U6NVY3 | 0.53 | 192392.9 | 5.69 | L_4_ |
|  | Myosin-4 | U6NXG6 | 1.26 | 80818.34 | 5.25 | L_4_ |
|  | SWIRM and Myb and XYPPX repeat domain containing protein | U6NXM1 | 0.63 | 102069.4 | 5.38 | L_4_ |
|  | Pre-mRNA-splicing factor 3 | U6NY38 | 1.08 | 75295.72 | 9.52 | L_4_ |
|  | Thyroglobulin type-1 and Proteinase inhibitor I2 | U6NZE8 | 0.29 | 225192.3 | 6.1 | L_4_ |
|  | scaffold_pathogens_Hcontortus_scaffold_234 | U6P0C4 | 3.13 | 35204.51 | 5.65 | L_4_ |
|  | Amine oxidase and Apyrase | U6P152 | 0.60 | 131979.1 | 6.98 | L_4_ |
|  | Hly-III related domain containing protein | U6P2D6 | 1.16 | 68307.16 | 5.62 | L_4_ |
|  | Heat shock protein DnaJ | U6P3E3 | 1.34 | 42182.78 | 6.5 | L_4_ |
|  | Scaffold_pathogens_Hcontortus_scaffold_2163 | U6P4H0 | 1.67 | 53582.65 | 6.37 | L_4_ |
|  | scaffold_pathogens_Hcontortus_scaffold_2802 | U6P503 | 0.87 | 90228.06 | 10.17 | L_4_ |
|  | Girdin isoform 2 | U6P8R2 | 1.00 | 78935.5 | 6.12 | L_4_ |
|  | Iron hydrogenase | U6P8W1 | 1.83 | 41985.06 | 5.97 | L_4_ |
|  | CBN-PES-8 protein | U6P8X5 | 3.48 | 36002.74 | 6.42 | L_4_ |
|  | Uncharacterized protein | U6PAF3 | 1.38 | 57742.41 | 6.22 | L_4_ |
|  | Methylated-DNA-[protein]-cysteine S-methyltransferase | U6PAP6 | 4.82 | 18409.21 | 9.04 | L_4_ |
|  | Aldehyde dehydrogenase | U6PAV3 | 1.95 | 62285.13 | 6.49 | L_4_ |
|  | Protein F44E2.3 | U6PB28 | 2.42 | 29522.71 | 10.01 | L_4_ |
|  | Ubiquitin carboxyl-terminal hydrolase | U6PC72 | 0.75 | 134456 | 6 | L_4_ |
|  | Protein F36A2.7 | U6PCN6 | 3.98 | 20799.42 | 9.15 | L_4_ |
|  | Kelch repeat type 1 and Kelch repeat type 2 | U6PCR9 | 0.98 | 68990.73 | 8.69 | L_4_ |
|  | scaffold_pathogens_Hcontortus_scaffold_3027 | U6PD16 | 4.84 | 14004.13 | 6.28 | L_4_ |
|  | Protein D2024.5, isoform a | U6PDL5 | 3.35 | 22087.83 | 9.05 | L_4_ |
|  | Uncharacterized protein | U6PEP9 | 0.37 | 185054.1 | 6.18 | L_4_ |
|  | Phox domain containing protein | U6PF25 | 2.53 | 44973.23 | 5.33 | L_4_ |
|  | Scaffold_pathogens_Hcontortus_scaffold_4255 (Fragment) | U6PF27 | 2.83 | 25233.64 | 9.74 | L_4_ |
|  | Uncharacterized protein | U6PF64 | 2.74 | 25240.87 | 8.96 | L_4_ |
|  | DNA-directed RNA polymerase | U6PFA6 | 0.38 | 204883.8 | 8.81 | L_4_ |
|  | GDPGTP exchange factor Sec2p domain containing protein | U6PFD5 | 2.28 | 39949.41 | 6.58 | L_4_ |
|  | Taxilin domain containing protein | U6PGD3 | 1.49 | 53863.32 | 9 | L_4_ |
|  | Scaffold_pathogens_Hcontortus_scaffold_4927 | U6PH19 | 1.22 | 64223.13 | 5.09 | L_4_ |
|  | Phosphoglycerate mutase | U6PH93 | 0.66 | 81789.54 | 9.62 | L_4_ |
|  | Uncharacterized protein | U6PHF4 | 1.52 | 53904.75 | 5.35 | L_4_ |
|  | MADF domain containing protein | U6PK17 | 2.08 | 38696.16 | 5.83 | L_4_ |
|  | Histone core domain | U6PL99 | 3.17 | 21113.21 | 10.57 | L_4_ |
|  | Scaffold_pathogens_Hcontortus_scaffold_5261 | U6PLR8 | 7.09 | 16002.39 | 9.64 | L_4_ |
|  | BRCT domain containing protein | U6PM41 | 1.70 | 45791.38 | 6.13 | L_4_ |
|  | Protein EGO-1 | U6PM45 | 1.75 | 46391.47 | 6.45 | L_4_ |
|  | scaffold_pathogens_Hcontortus_scaffold_566 | U6PNC7 | 6.59 | 18169.21 | 10.09 | L_4_ |
|  | Protein TAG-333, isoform a | U6PNF6 | 1.14 | 67986.53 | 8.94 | L_4_ |
|  | Protein SZT2-like (Fragment) | U6PP26 | 0.60 | 113904 | 7.1 | L_4_ |
|  | Glucose-methanol-choline oxidoreductase and Eukaryotic translation initiation factor 3 | U6PPD1 | 0.82 | 95351.93 | 8.67 | L_4_ |
|  | DNA RNA helicase | U6PPH1 | 5.94 | 24511.41 | 8.18 | L_4_ |
|  | BTB/POZ domain-containing protein 8 | U6PPH4 | 4.84 | 33890.68 | 5.28 | L_4_ |
|  | scaffold_pathogens_Hcontortus_scaffold_621 | U6PQ96 | 2.26 | 44231.82 | 9.86 | L_4_ |
|  | Scaffold_pathogens_Hcontortus_scaffold_636 |  |  |  |  | L_4_ |
|  | Uncharacterized protein | U6PQX7 | 2.55 | 26589.67 | 8.98 | L_4_ |
|  | SET domain containing protein | U6PR04 | 1.76 | 45748.67 | 4.96 | L_4_ |
|  | Cullin and Cullin protein domain containing protein | U6PT65 | 0.81 | 100493.1 | 8.69 | L_4_ |
|  | Uncharacterized protein | U6PTI5 | 8.91 | 11291.28 | 6.04 | L_4_ |
|  | Neurotransmitter-gated ion-channel ligand-binding and Neurotransmitter-gated ion-channel transmembrane region | U6PU50 | 1.34 | 60753.63 | 8.4 | L_4_ |
|  | WD repeat and HMG-box DNA-binding protein 1 | U6PV63 | 2.69 | 61650.32 | 5.27 | L_4_ |
|  | Scaffold_pathogens_Hcontortus_scaffold_793 | U6PVB7 | 2.45 | 32743.13 | 5.78 | L_4_ |
|  | Uncharacterized protein | U6PVU2 | 0.59 | 131942.2 | 7.89 | L_4_ |
|  | Uncharacterized protein | U6PXR0 | 1.52 | 68049.99 | 9.21 | L_4_ |
|  | Proteophosphoglycan ppg1 | U6PXY4 | 0.40 | 194152.2 | 9.22 | L_4_ |
|  | Structural maintenance of chromosomes protein | U6PZP5 | 0.55 | 165168.7 | 6.21 | L_4_ |
|  | Uncharacterized protein | U6Q0H0 | 0.59 | 112973.4 | 6.39 | L_4_ |
|  | Bardet-Biedl syndrome 1 protein isoform 2 | W6NDG1 | 3.35 | 26387.17 | 6.2 | L_4_ |
|  | Uso1 p115 like vesicle tethering protein | W6NDN2 | 3.29 | 35087.78 | 5.33 | L_4_ |
|  | ISE/inbred ISE, contig Hcontortus_contig_pathogens_Hcontortus_scaffold_5814 | W6NEX5 | 4.93 | 15826.8 | 5.39 | L_4_ |
|  | Subtilisin- like serine protease | W6NS46 | 0.62 | 89094.47 | 6.05 | L_4_ |
|  | Dynamitin domain containing protein | W6NV29 | 2.65 | 38141.59 | 5.1 | L_4_ |
|  | scaffold_pathogens_Hcontortus_scaffold_17 | U6NUV5 | 3.21 | 27937.86 | 9.92 | L_5_ |
|  | Telomerase activating protein Est1 | U6NEU1 | 1.14 | 148292.9 | 6.25 | L_5_ |
|  | Enolase | U6PW36 | 2.77 | 47075.2 | 6.81 | L_5_ |
|  | ISE/inbred ISE, contig Hcontortus_contig_pathogens_Hcontortus_scaffold_11668 | W6NI14 | 15.38 | 16779.1 | 9.72 | L_5_ |
|  | Uncharacterized protein | U6P3W1 | 0.90 | 62510.07 | 9.24 | L_5_ |
|  | scaffold_pathogens_Hcontortus_scaffold_475 | U6PJK8 | 2.52 | 40191.81 | 10.3 | L_5_ |
|  | Unc- 74 protein | G0XFD8 | 2.02 | 50667.21 | 5.11 | L_5_ |
|  | CBN-FLR-4 protein | U6NF84 | 7.89 | 8877.03 | 6.27 | L_5_ |
|  | Uncharacterized protein | U6NH21 | 8.02 | 18941.49 | 8.6 | L_5_ |
|  | EGF and Hyalin and GCC2 GCC3 domain containing protein | U6NLI6 | 0.45 | 146238.6 | 5.46 | L_5_ |
|  | Uncharacterized protein | U6NLX1 | 0.78 | 100079.8 | 6.2 | L_5_ |
|  | Nrap protein | U6NMQ0 | 0.64 | 158241.8 | 6.78 | L_5_ |
|  | Protein NSY-7, isoform b | U6NMU3 | 3.01 | 29483.48 | 5.19 | L_5_ |
|  | CBN-CRTC-1 protein | U6NN63 | 2.16 | 35376.26 | 5.93 | L_5_ |
|  | Signal recognition particle receptor and Signal recognition particle | U6NNL2 | 0.91 | 72811.03 | 8.48 | L_5_ |
|  | MAD homology 1 and SMAD domain containing protein | U6NNM6 | 1.59 | 56536.17 | 6.5 | L_5_ |
|  | Saposin type B | U6NNX7 | 7.77 | 11344.3 | 5.9 | L_5_ |
|  | Uncharacterized protein | U6NQ56 | 2.79 | 29335.35 | 9.38 | L_5_ |
|  | COG complex component | U6NQA1 | 0.96 | 81533.52 | 5.56 | L_5_ |
|  | scaffold_pathogens_Hcontortus_scaffold_1217 | U6NR14 | 3.33 | 23350.97 | 5.92 | L_5_ |
|  | Uncharacterized protein | U6NRE8 | 3.45 | 22845.13 | 9.15 | L_5_ |
|  | Ion transport 2 | U6NRL0 | 1.16 | 58873.33 | 8.55 | L_5_ |
|  | Fps Fes Fer CIP4 homology and Src homology-3 | U6NRP7 | 1.72 | 52669.69 | 6.52 | L_5_ |
|  | Carbohydrate  kinase | U6NRV6 | 1.91 | 45995.94 | 6.76 | L_5_ |
|  | Acyltransferase ChoActase COT CPT | U6NRY4 | 0.60 | 116638.5 | 8.27 | L_5_ |
|  | Uncharacterized protein | U6NT62 | 0.71 | 145845.1 | 6.19 | L_5_ |
|  | Alkyl hydroperoxide reductase Thiol specific antioxidant Mal allergen and Peroxiredoxin | U6NTW3 | 3.52 | 22301.37 | 6.42 | L_5_ |
|  | Immunoglobulin | U6NTX7 | 2.30 | 29570.43 | 6.21 | L_5_ |
|  | Uncharacterized protein | U6NU82 | 0.39 | 176598 | 8.84 | L_5_ |
|  | Anaphase-promoting complex | U6NVU8 | 2.79 | 24504.42 | 6.24 | L_5_ |
|  | Uncharacterized protein | U6NWC2 | 0.97 | 119988.2 | 4.97 | L_5_ |
|  | Uncharacterized protein | U6NWZ3 | 0.56 | 259138.9 | 6.26 | L_5_ |
|  | Fanconi anemia group I protein-like | U6NXQ1 | 0.70 | 113620.9 | 6.46 | L_5_ |
|  | DNA replication factor CDT1 domain containing protein | U6NYF6 | 0.68 | 83076.8 | 9.6 | L_5_ |
|  | Phosphotyrosyl phosphatase activator domain containing protein | U6NYR3 | 1.83 | 38317.93 | 4.81 | L_5_ |
|  | Tensin phosphotyrosine-binding and Variant SH3 | U6NZB3 | 0.78 | 101582.2 | 6.83 | L_5_ |
|  | Uncharacterized protein | U6P049 | 2.29 | 34783.6 | 6.74 | L_5_ |
|  | Myosin-10 | U6P075 | 1.02 | 88162.57 | 5.75 | L_5_ |
|  | Ephrin | U6P1E3 | 4.18 | 29769.15 | 5.16 | L_5_ |
|  | HAT dimerisation domain containing protein | U6P1I0 | 0.82 | 94509.01 | 5.91 | L_5_ |
|  | Uncharacterized protein | U6P1Z9 | 3.86 | 24177.6 | 6.26 | L_5_ |
|  | Bestrophin domain containing protein | U6P358 | 1.38 | 58756.21 | 5.62 | L_5_ |
|  | Uncharacterized protein | U6P3P5 | 0.81 | 84172.2 | 8.68 | L_5_ |
|  | Basic helix-loop-helix dimerisation region bHLH | U6P4V6 | 3.91 | 20757.03 | 9.58 | L_5_ |
|  | Protein ALP-1, isoform b | U6P6T8 | 7.69 | 18704.6 | 5.18 | L_5_ |
|  | Glutamine amidotransferase | U6P6W8 | 1.80 | 49884.13 | 5.74 | L_5_ |
|  | Uncharacterized protein | U6P701 | 2.60 | 37224.89 | 4.84 | L_5_ |
|  | Protein C49C3.4 | U6P7H6 | 1.16 | 160847.7 | 5.03 | L_5_ |
|  | Bicaudal-D protein domain containing protein | U6P7S6 | 0.75 | 90441.97 | 4.99 | L_5_ |
|  | Uncharacterized protein | U6P7Z0 | 1.33 | 50073.58 | 9.6 | L_5_ |
|  | Gamma-glutamyltranspeptidase | U6P899 | 0.94 | 70413.81 | 5.73 | L_5_ |
|  | CBN-PES-8 | U6P8X5 | 3.48 | 36002.74 | 6.42 | L_5_ |
|  | Cellular retinaldehyde-binding triple function domain containing protein | U6PAD7 | 2.07 | 44051.31 | 6.74 | L_5_ |
|  | Scaffold_pathogens_Hcontortus_scaffold_2777 | U6PAL8 | 6.80 | 11695.29 | 6.88 | L_5_ |
|  | Uncharacterized protein | U6PAS0 | 0.84 | 82747.79 | 8.49 | L_5_ |
|  | Reduced folate carrier domain containing protein | U6PB12 | 3.60 | 49900.03 | 6.39 | L_5_ |
|  | Gag-Pol polyprotein | U6PB44 | 2.79 | 41700.52 | 9.13 | L_5_ |
|  | Protein F36A2.7 | U6PCN6 | 3.98 | 20799.42 | 9.15 | L_5_ |
|  | Scaffold_pathogens_Hcontortus_scaffold_412 | U6PD14 | 3.92 | 23539.3 | 8.37 | L_5_ |
|  | Emp24 gp25L p24 domain containing protein | U6PDD1 | 3.02 | 26281.92 | 5.97 | L_5_ |
|  | Magnesium transporter and Squalene phytoene synthase | U6PDW9 | 0.31 | 255179.6 | 5.59 | L_5_ |
|  | Protein PERM-5 | U6PEL3 | 1.42 | 55138.67 | 5.11 | L_5_ |
|  | Leucine Rich Repeat family protein | U6PGY0 | 0.89 | 87717.01 | 5.73 | L_5_ |
|  | Peptidyl-prolyl cis-trans isomerase | U6PH31 | 5.45 | 21708.38 | 6.07 | L_5_ |
|  | Uncharacterized protein | U6PHD5 | 4.76 | 17005.37 | 10.51 | L_5_ |
|  | Aromatic amino acid beta-eliminating lyase threonine aldolase | U6PHL6 | 1.76 | 43817.82 | 8.11 | L_5_ |
|  | Scaffold_pathogens_Hcontortus_scaffold_4435 | U6PI31 | 4.46 | 18188.61 | 9.49 | L_5_ |
|  | Phytanoyl-CoA hydroxylase-interacting | U6PK30 | 1.25 | 64741.27 | 6.04 | L_5_ |
|  | Taurine catabolism dioxygenase TauD TfdA domain | U6PKQ7 | 1.43 | 48354.49 | 6.57 | L_5_ |
|  | Leucine-rich repeat and Gelsolin region domain containing protein | U6PKY5 | 0.99 | 103787.5 | 5.6 | L_5_ |
|  | scaffold_pathogens_Hcontortus_scaffold_5261 | U6PLR8 | 7.09 | 16002.39 | 9.64 | L_5_ |
|  | Scaffold_pathogens_Hcontortus_scaffold_533 | U6PM03 | 3.27 | 25262.36 | 9.75 | L_5_ |
|  | Chemotaxis methyl-accepting receptor signaling | U6PMV4 | 0.79 | 102468.6 | 5.99 | L_5_ |
|  | C2 calcium-dependent membrane targeting domain containing protein | U6PMX4 | 2.68 | 37943.94 | 7.65 | L_5_ |
|  | ATP dependent DNA ligase and BRCT domain containing protein | U6PPC2 | 0.70 | 130351.9 | 8.4 | L_5_ |
|  | Protein NHR-6, isoform b | U6PPD4 | 1.84 | 36535.66 | 8.19 | L_5_ |
|  | Uncharacterized protein | U6PQ04 | 0.14 | 571660.4 | 5.08 | L_5_ |
|  | Mitochondrial inner membrane protein Mitofilin | U6PQ62 | 1.63 | 75791.64 | 6.33 | L_5_ |
|  | Emp24 gp25L p24 domain containing protein | U6PQC4 | 3.38 | 23926.46 | 7.7 | L_5_ |
|  | Scaffold_pathogens_Hcontortus_scaffold_74 | U6PQJ5 | 5.39 | 18877.2 | 9.64 | L_5_ |
|  | Miro domain containing protein | U6PQV2 | 2.69 | 33688.14 | 6.4 | L_5_ |
|  | Small-subunit processome | U6PR51 | 0.97 | 81949.4 | 9.14 | L_5_ |
|  | Major facilitator superfamily protein | U6PS28 | 1.70 | 58271.1 | 5.17 | L_5_ |
|  | Filament and Intermediate filament domain containing protein | U6PS36 | 1.97 | 64205.3 | 5.63 | L_5_ |
|  | Scaffold_pathogens_Hcontortus_scaffold_567 | U6PS86 | 2.00 | 45611.13 | 5.46 | L_5_ |
|  | Scaffold_pathogens_Hcontortus_scaffold_704 | U6PV41 | 1.03 | 87512.07 | 5.52 | L_5_ |
|  | Scaffold_pathogens_Hcontortus_scaffold_664 | U6PVG0 | 1.30 | 65897.02 | 11.04 | L_5_ |
|  | Uncharacterized protein | U6PWS1 | 10.38 | 10666.27 | 9 | L_5_ |
|  | Transmembrane channel-like protein 5 | U6PX75 | 2.65 | 65233.06 | 5.11 | L_5_ |
|  | Uncharacterized protein | U6PXZ2 | 1.56 | 48904.26 | 6.59 | L_5_ |
|  | Electron transfer flavoprotein | U6PZL6 | 2.11 | 34518.24 | 8.91 | L_5_ |
|  | Annexin | U6PZT1 | 2.07 | 38026.87 | 6.56 | L_5_ |
|  | Uncharacterized protein | U6Q028 | 3.38 | 27432.56 | 8.45 | L_5_ |
|  | Scaffold_pathogens_Hcontortus_scaffold_989 | U6Q0C6 | 3.57 | 29489.4 | 9.13 | L_5_ |
|  | Uncharacterized protein | U6Q0J4 | 0.90 | 100975.7 | 6.59 | L_5_ |
|  | Transcription factor TFIIIB component B | W6NA54 | 0.89 | 87792.16 | 5.49 | L_5_ |
|  | Uncharacterized protein | W6NAP7 | 2.45 | 37613.37 | 9.05 | L_5_ |
|  | Hcontortus_contig_pathogens_Hcontortus_scaffold_1735 | W6NAS1 | 0.89 | 87477.4 | 10.76 | L_5_ |
|  | Inorganic pyrophosphatase domain containing protein | W6NCB2 | 2.76 | 32547.67 | 5.82 | L_5_ |
|  | Lipid transport protein domain containing protein | W6NCY7 | 6.28 | 24490.72 | 5.03 | L_5_ |
|  | COG4 transport domain containing protein | W6NF34 | 0.68 | 99020.47 | 5.39 | L_5_ |
|  | Hcontortus_contig_pathogens_Hcontortus_scaffold_3330 | W6NFM5 | 3.11 | 42490.73 | 9.86 | L_5_ |
|  | Hcontortus_contig_pathogens_Hcontortus_scaffold_3720 | W6NG86 | 4.49 | 17878.47 | 4.71 | L_5_ |
|  | Hcontortus_contig_pathogens_Hcontortus_scaffold_7470 | W6NHX8 | 0.90 | 97891.37 | 7.69 | L_5_ |
|  | Protein T05C1.3 | W6NHY8 | 1.67 | 67323.38 | 5.85 | L_5_ |
|  | Innexin | W6NID6 | 1.82 | 44958.74 | 7.17 | L_5_ |
|  | Histone H1 H5 | W6NN22 | 3.78 | 26551.32 | 11.06 | L_5_ |
|  | Uncharacterized protein | W6NVM2 | 2.71 | 29517.36 | 5.2 | L_5_ |
|  | Protein UNC-44, isoform | U6NWN1 | 0.99 | 112028.5 | 4.19 | Early adult |
|  | Scaffold_pathogens_Hcontortus_scaffold_5392 | U6PR33 | 3.88 | 15384.38 | 5.17 | Early adult |
|  | Amino acid transporter domain containing protein | U6PYI8 | 2.17 | 51365.15 | 8.97 | Early adult |
|  | CBN-GRK-1 protein (Fragment) | U6PGK4 | 6.96 | 17918.33 | 8.46 | Early adult |
|  | Alanine racemase | U6P9P5 | 3.03 | 18661.37 | 6.42 | Early adult |
|  | Condensation and AMP-dependent synthetase ligase | U6PHV3 | 0.73 | 139486.8 | 6.2 | Early adult |
|  | Scaffold_pathogens_Hcontortus_scaffold_130 | U6NK96 | 12.82 | 8840.73 | 4.57 | Early adult |
|  | Scaffold_pathogens_Hcontortus_scaffold_1284 | U6NS68 | 6.06 | 11400.94 | 5.19 | Early adult |
|  | Aminotransferase | U6NVC5 | 2.20 | 44352.55 | 8.35 | Early adult |
|  | Protein Y79H2A.3, isoform b | U6P270 | 1.05 | 131519.1 | 8.64 | Early adult |
|  | Uncharacterized protein | U6PCK4 | 1.11 | 61473.59 | 5.24 | Early adult |
|  | PHD finger protein 3-like | U6PK09 | 0.39 | 194515.7 | 5.62 | Early adult |
|  | BRCT domain containing protein | U6PM41 | 1.70 | 45791.38 | 6.13 | Early adult |
|  | CK1/WORM6 protein kinase | W6NFI2 | 1.69 | 67517.06 | 9.59 | Late adult |
|  | Uncharacterized protein | W6NXF3 | 3.97 | 17858.11 | 6.84 | Late adult |
|  | Protein synthesis factor and Translation elongation factor EFTu EF1A and Translation elongation factor EFG EF2 | U6PV91 | 1.88 | 94526.1 | 6.17 | Late adult |
|  | Selectin-like protein | U6NW15 | 1.19 | 92401.59 | 4.7 | Late adult |
|  | PDZ domain | U6P1S1 | 0.24 | 222601.4 | 6.07 | Late adult |
|  | Protein B0403.5 | U6NXD5 | 4.20 | 26045.01 | 5.05 | Late adult |
|  | Uncharacterised protein family UPF0171 | U6PD81 | 0.92 | 60728.3 | 6.77 | Late adult |
|  | TFIIH p62 subunit and BSD | U6PF34 | 0.82 | 70073.31 | 5.21 | Late adult |
|  | Protein T16G12.9, isoform a | W6NG25 | 3.05 | 36593.49 | 6.65 | Late adult |
|  | Maternal tudor protein | U6PBT4 | 2.54 | 44128.22 | 7.91 | Late adult |
|  | Uncharacterized protein | U6PFC0 | 1.24 | 72128.69 | 5.68 | Late adult |
|  | Protein LIN-13 | U6PWJ2 | 0.53 | 230700.8 | 6.75 | Late adult |
|  | GF receptor domain containing protein | U6NS04 | 0.99 | 93271.18 | 6.13 | Late adult |
|  | Conserved oligomeric complex COG6 | U6PEV5 | 1.08 | 73219.84 | 5.37 | Late adult |
|  | DNA RNA helicase domain | U6PPH1 | 5.94 | 24511.41 | 8.18 | Late adult |
|  | Protein F52C9.7 | U6PWC3 | 2.29 | 50585.87 | 9.63 | Late adult |
|  | Protein MDT-9, isoform a | U6PZW9 | 11.32 | 12326.74 | 5.2 | Late adult |
|  | Short-chain dehydrogenase reductase SDR | W6NF84 | 2.30 | 27533.42 | 8.4 | Late adult |
|  | Uncharacterized protein | W6NVE0 | 6.09 | 13084.64 | 9.2 | Late adult |
|  | Protein F13B9.2 | U6NHX8 | 1.53 | 77650.13 | 6.61 | Late adult |
|  | Regulator of nonsense transcripts 1 homolog | U6NQ08 | 0.70 | 141617.9 | 8.54 | Late adult |
|  | Uncharacterized protein | U6P0H7 | 2.61 | 46514.81 | 9.66 | Late adult |
|  | 5-hydroxyisourate hydrolase | U6P6I7 | 4.41 | 15367.69 | 9.23 | Late adult |
|  | Scaffold_pathogens_Hcontortus_scaffold_364 | U6PBG6 | 4.43 | 17317.59 | 5.36 | Late adult |
|  | Uncharacterized protein | U6PII2 | 2.54 | 39345.76 | 4.79 | Late adult |
|  | Scaffold_pathogens_Hcontortus_scaffold_5996 | U6PME7 | 15.74 | 12979.76 | 4.7 | Late adult |
|  | Uncharacterized protein | W6NR83 | 1.62 | 48313.84 | 5.99 | Late adult |
|  | Histidine acid phosphatase domain containing protein | U6NG75 | 2.36 | 39056.25 | 8.19 | Late adult |
|  | Uncharacterized protein | U6NN21 | 2.88 | 32017.51 | 9.36 | Late adult |
|  | Acidic leucine-rich nuclear phosphoprotein 32 | U6NTB2 | 4.76 | 25204.39 | 4.22 | Late adult |
|  | ACL-8 protein | U6NTK4 | 4.05 | 25869.81 | 6.52 | Late adult |
|  | Scaffold_pathogens_Hcontortus_scaffold_151 | U6NTS3 | 1.25 | 54180.42 | 10.13 | Late adult |
|  | Uncharacterized protein LOC100840703 | U6NUF1 | 6.96 | 13721.93 | 10.32 | Late adult |
|  | Hypothetical 96.7 kDa protein C32D5.3 in | U6NZ09 | 1.65 | 95551.47 | 5.91 | Late adult |
|  | Protein EVL-14 | U6P0G7 | 0.70 | 177189.6 | 9.13 | Late adult |
|  | Leucine-rich repeat and PDZ | U6P1T1 | 0.50 | 153062.4 | 5.4 | Late adult |
|  | Scaffold_pathogens_Hcontortus_scaffold_25 | U6P1W8 | 4.44 | 31494.65 | 5.77 | Late adult |
|  | Protein synthesis factor | U6P2B5 | 3.06 | 32225.96 | 4.9 | Late adult |
|  | Golgi autoantigen, golgin subfamily a, 4 | U6PT55 | 0.34 | 231651.4 | 4.87 | Late adult |
|  | Transcription factor jumonji 1 | U6PYV8 | 1.39 | 49339.55 | 8.23 | Late adult |
|  | Transcription factor jumonji domain containing protein | U6Q0J3 | 2.10 | 59989.53 | 5.01 | Late adult |
|  | ISE/inbred ISE, contig Hcontortus_contig_pathogens_Hcontortus_scaffold_3978 | W6ND00 | 8.29 | 22731.43 | 8.92 | Late adult |
|  | Ankyrin and Sad1 UNC and Mib-herc2 and HECT | W6NDE6 | 0.23 | 287613.4 | 5.32 | Late adult |
|  | DNA RNA helicase domain | W6NF68 | 1.30 | 61039.48 | 9.03 | Late adult |
|  | Myosin heavy chain | W6NG12 | 1.22 | 92378.37 | 5.03 | Late adult |
|  | Uncharacterized protein | W6NRG8 | 0.61 | 149104.6 | 6.25 | Late adult |
|  | Tyrosine protein kinase | U6NSF2 |  |  |  | Late adult |
