# Supplementary material for: Proteomic Analysis of the Excretory and Secretory Proteins of Haemonchus contortus (HcESP) Binding to Goat PBMCs In Vivo Revealed Stage-Specific Binding Profiles
Source: PLoS One. 2016 Jul 28;11(7):e0159796. doi: 10.1371/journal.pone.0159796 (PMC4965049; doi:10.1371/journal.pone.0159796)
Supplement: S3 Table — (DOCX) [file pone.0159796.s004.docx]

**S3 Table:** List of proteins available on string database matched with our Query sequences of unassigned proteins analyzed for Functional annotation improvement by protein clustering analysis

| **Sr. NO.** | **Query sequence ID** | **STRING protein Description** | **Identity %** | **Developmental stages** |
| --- | --- | --- | --- | --- |
|  | U6PBJ7 | arf-1.2 | 97 | L_4_, L_5_ and late adult |
|  | U6PP12 | ftt-2 | 93 | L_4_, L_5,_ early and late adult |
|  | W6NEU0 | arx-2 | 90 | L_4_, L_5,_ early and late adult |
|  | 'U6PTS0 | act-2 | 87 | L_4_, L_5_ early and late adult |
|  | \|U6PH81 | arx-1 | 86 | L_4_, L_5,_ early and late adult |
|  | U6PS86 | acc-4 | 86 | L_5_ |
|  | U6PMX4 | snt-4 | 84 | L_5_ |
|  | W6NXF3 | K02B2.3 | 82 | Late adult |
|  | U6PKY5 | fli-1 | 77 | L_5_ |
|  | U6PXR0 | C09F12.2 | 75 | L_4_ |
|  | U6P358 | ZK688.2 | 73 | L_5_ |
|  | U6NQ56 | C11H1.9 | 72 |  |
|  | U6P1W8 | ncbp-2 | 71 | Late adult |
|  | U6PF27 | lgc-49 | 71 | L_4_ |
|  | U6PH16 | lev-11 | 71 | L_4_ and L_5_ |
|  | U6PN49 | gop-1 | 70 | L_4_ |
|  | U6NLN1 | vps-36 | 69 | L_4_ |
|  | U6P6T8 | alp-1 | 69 | L_5_ |
|  | U6NZ09 | C32D5.3 | 68 | Late adult |
|  | U6PQX7 | tag-76 | 67 | L_4_ |
|  | U6PCN6 | F36A2.7 | 66 | L_4_ |
|  | W6NHX8 | pgp-9 | 66 | L_5_ |
|  | U6NSU7 | sax-7 | 65 | L_4_ and L_5_ |
|  | U6NXD5 | B0403.5 | 65 | Late adult |
|  | U6NPE8 | nlp-16 | 64 | L_4_ |
|  | U6P3E3 | dnj-16 | 63 | L_4_ |
|  | U6PFD5 | F54C9.11 | 62 | L_4_ |
|  | U6NH21 | C10F3.7 | 60 | L_5_ |
|  | U6NTH3 | cpsf-2 | 60 | L_4_, L_5_ and early adult |
|  | U6PWC3 | mog-3 | 60 | Late adult |
|  | U6PHD5 | ZK1236.5 | 59 | L_5_ |
|  | U6PWS1 | W01A8.2 | 59 | L_5_ |
|  | U6NIA2 | pqn-87 | 58 | L_4_ |
|  | U6NRE8 | C07A9.12 | 58 | L_5_ |
|  | U6NVU8 | Y48G1C.12 | 58 | L_5_ |
|  | U6P1S1 | mpz-1 | 57 | Late adult |
|  | U6NP15 | wht-8 | 56 | L_4_ and L_5_ |
|  | U6NN67 | F32E10.8 | 55 | L_4_ |
|  | U6NSZ7 | C37A2.6 | 55 | L_4_ |
|  | U6P075 | B0432.7 | 55 | L_5_ |
|  | U6NTX7 | lad-2 | 54 | L_5_ |
|  | U6NZB3 | eps-8 | 54 | L_5_ |
|  | U6P8X5 | pes-8 | 54 | L_4_ |
|  | U6NSR1 | ZC395.10 | 53 | L_5_  and Late adult |
|  | U6P9P5 | F09E5.8 | 53 | Early adult |
|  | U6PGD3 | T22C1.6 | 53 | L_4_ |
|  | U6Q0J3 | psr-1 | 53 | Late adult |
|  | U6NL27 | K01A2.10 | 52 | Early and late adult |
|  | U6PB28 | F44E2.3 | 52 | L_4_ |
|  | U6NKJ3 | C11D9.1 | 50 | L_4_ |
|  | U6NNX7 | spp-3 | 50 | L_5_ |
|  | U6NTK4 | acl-9 | 50 | Late adult |
|  | U6P1T1 | let-413 | 50 | Late adult |
|  | U6PEP9 | F46B6.12 | 50 | L_4_ |
|  | U6NLX1 | vps-54 | 48 | L_5_ |
|  | U6NWZ3 | C56C10.12 | 48 | L_5_ |
|  | U6P8R2 | Y51A2D.15 | 48 | L_4_ |
|  | W6NF34 | cogc-4 | 48 | L_5_ |
|  | W6NVM2 | ZK1307.9 | 48 | L_5_ |
|  | U6NWN1 | unc-44 | 47 | Early adult |
|  | U6NZK6 | unc-82 | 47 | L_4_, L_5_ and late adult |
|  | U6PGY0 | zyg-11 | 47 | L_5_ |
|  | U6NF84 | flr-4 | 46 | L_5_ |
|  | U6NRP7 | sdpn-1 | 46 | L_5_ |
|  | U6NTB2 | F33H2.3 | 46 | Late adult |
|  | U6PR04 | set-29 | 45 | L_4_ |
|  | U6NN63 | Y20F4.2 | 44 | L_5_ |
|  | U6P4V6 | hlh-10 | 44 | L_5_ |
|  | U6P8W1 | oxy-4 | 44 | L_4_ |
|  | U6PYV8 | F29B9.2 | 43 | Late adult |
|  | U6NMU3 | nsy-7 | 42 | L_5_ |
|  | U6P701 | F53H2.3 | 42 | L_5_ |
|  | U6NXG6 | F46H5.7 | 41 | L_4_ |
|  | U6PP26 | F54B3.1 | 41 | L_4_ |
|  | U6NN21 | K07F5.16 | 40 | Late adult |
|  | U6PAI2 | F31F7.2 | 40 | L_4_, L_5_ and early adult |
|  | U6NYF6 | cdt-1 | 39 | L_5_ |
|  | U6PD81 | F35H10.7 | 39 | Late adult |
|  | U6P5P5 | ric-8 | 38 | L_4_  and L_5_ |
|  | U6PDL5 | D2024.5 | 38 | L_4_ |
|  | W6NVE0 | F59A2.6 | 38 | Late adult |
|  | U6PNF6 | tag-333 | 36 | L_4_ |
|  | W6NH38 | ZK1055.7 | 36 | L_4_ |
|  | U6PK30 | Y92H12BR.7 | 35 | L_5_ |
|  | U6NK13 | F13E9.14 | 34 | L_4_ |
|  | U6NMU2 | zim-1 | 34 | L_4_ |
|  | U6NNG2 | C27D8.3 | 34 | L_4_ |
|  | U6PH93 | F55A11.11 | 34 | L_4_ |
|  | U6NT62 | ZC132.6 | 33 | L_5_ |
|  | U6NXQ1 | fnci-1 | 33 | L_5_ |
|  | U6P0H7 | K07A1.3 | 33 | L_4_ and Late adult |
|  | U6PLW2 | ZK596.1 | 33 | L_4_ |
|  | U6PRY3 | anc-1 | 33 | L_4_ and L_5_ |
|  | U6PXY4 | F54D11.2 | 32 | L_4_ |
|  | W6NRG8 | Y92H12A.5 | 32 | Late adult |
|  | U6NEU1 | smg-6 | 31 | L_5_ |
|  | U6P270 | C51G7.3 | 31 | Early adult |
|  | U6PCR9 | Y50D7A.1 | 31 | L_4_ |
|  | U6PII4 | srr-1 | 31 | L_5_, early and late adult |
|  | U6PQ04 | cpna-2 | 30 | L_5_ |
|  | U6PYX6 | ndc-80 | 30 | L_4_ and late adult |
|  | W6NFM5 | sst-20 | 30 | L_5_ |
|  | U6NM23 | B0511.11 | 29 | L_4_ |
|  | U6P0G7 | evl-14 | 29 | Late adult |
|  | U6P1Z9 | C28C12.4 | 29 | L_5_ |
|  | U6P7H6 | C49C3.4 | 28 | L_5_ |
|  | U6PMV4 | F59A6.5 | 28 | L_5_ |
|  | U6PP16 | Y75B8A.8 | 28 | L_5_  and late adult |
|  | W6NG25 | T16G12.9B | 28 | Late adult |
|  | U6NI50 | lin-61 | 27 | L_5_ and early adult |
|  | U6PAF3 | Y54E5A.2 | 27 | L_4_ |
|  | U6NQ08 | C05C10.2 | 26 | Late adult |
|  | U6NWC2 | T07C4.10 | 25 | L_5_ |
|  | U6PWJ2 | lin-13 | 24 | late adult |
|  | W6NAP7 | F55H2.7 | 24 | L_5_ |
|  | U6NYV8 | vap-1 | 23 | L_4_, L_5_ and late adult |
|  | U6PAS0 | Y50D4A.1 | 23 | L_5_ |
|  | U6PM41 | W04A8.1 | 23 | Early adult |
